# Working memory and attention in choice

**DOI:** 10.1371/journal.pone.0284127

**Published:** 2023-10-11

**Authors:** Aldo Rustichini, Philippe Domenech, Claudia Civai, Colin G. DeYoung

**Affiliations:** 1 Department of Economics, University of Minnesota, Hanson Hall, Minneapolis, MN, United States of America; 2 Neurosurgery Department, Henri Mondor Hospital, Paris, France, and Brain & Spine Institute, AP-HP, DHU PePsy, CRICM, CNRS UMR, Créteil, France; 3 Division of Psychology, School of Applied Sciences, London South Bank University, London, United Kingdom; 4 Department of Psychology, University of Minnesota, Elliott Hall, Minneapolis, MN, United States of America; University of Pisa, ITALY

## Abstract

We study the role of attention and working memory in choices where options are presented sequentially rather than simultaneously. We build a model where a costly attention effort is chosen, which can vary over time. Evidence is accumulated proportionally to this effort and the utility of the reward. Crucially, the evidence accumulated decays over time. Optimal attention allocation maximizes expected utility from final choice; the optimal solution takes the decay into account, so attention is preferentially devoted to later times; but convexity of the flow attention cost prevents it from being concentrated near the end. We test this model with a choice experiment where participants observe sequentially two options. In our data the option presented first is, everything else being equal, significantly less likely to be chosen. This *recency effect* has a natural explanation with appropriate parameter values in our model of leaky evidence accumulation, where the decline is stronger for the option observed first. Analysis of choice, response time and brain imaging data provide support for the model. Working memory plays an essential role. The recency bias is stronger for participants with weaker performance in working memory tasks. Also activity in parietal areas, coding the stored value in working, declines over time as predicted.

## Introduction

Economic models of choice usually abstract from the temporal dimension of the process that produces choice. An undesirable implication of this omission is the absence of any role of memory in theories of how choice is produced. Unfortunately, this omission is significant: in real life an individual rarely observes the available options presented at the same time. Recent research in economics, both applied and theoretical, has highlighted the potential effect of memory on choice. While these models are insightful, they do not even specify the type of memory (for example, working memory or long-term memory) that is the object of study. This seems an important limitation, that this study addresses. We find that introducing memory may significantly affect choice, and so should not be ignored.

### Working memory

The theory of memory poses a fundamental distinction between working memory and long-term memory; here we only consider the first. Working memory has three fundamental distinguishing characteristics: a substantial *constraint* on the amount of information that can be stored, the *decay* over time of this information, and the *availability* of the information for cognitive processing. The information in working memory can be processed, as is the case, for example, when we are asked to add numbers presented verbally in sequence. On the contrary, size constraints have little or no importance for long-term memory, and persistence of the information, which is based on protein synthesis, is considerably stronger. But information in long-term memory cannot be actively processed.

The two types of memory are linked in choice because the information stored in long-term memory has to be retrieved into working memory to be processed. This implies that working memory is *always* involved in choice, even when the relevant information is originally stored in long-term memory. Consider for example the choice among important alternatives in professional life (such as, in academia, changing a department of affiliation, or as students in choosing the PhD granting institution). In this case the relevant information on the alternatives (on characteristics such as the path of future compensations, opportunity for promotion, research environment and so on) is obviously stored in long-term memory. But this information has to be retrieved into working memory to be available for the information processing that produces choice. Choosing is difficult in part because the bottleneck of the size constraint of working memory is always present.

A proper consideration of these three features is missing from existing models of memory and economic choice. For example, models such as [1,2] focus on size constraint, and thus should more naturally be interpreted as models of working memory. However, this research takes into account only one of the three fundamental characteristics of working memory, the size constraint, and ignores both the decay and the availability for information processing. In particular, ignoring the decay of information is a very restrictive assumption, since decay directly affects choice behavior.

### Attention provision and evidence decay

Here we first develop a general theory of choice when options become available to the decision maker (*DM*) in a sequential way. Our experimental design is an instance of such situation. In our theory, the *DM* has to decide how much attention he wants to devote to each option, gathering evidence on each. Attention is costly, and evidence gathered decays with time. Our setup is general enough to include several interesting special cases of sequential choice, including the simultaneous presentation of the two options. In our experiment, participants observe two options (which are time delayed payments) sequentially. The time lag between the two presentations is short and randomized. After another random delay, a third display presents the two options together, and the participant is asked to choose one of the two. We observe choice behavior, time to respond and brain activation associated with these events.

### Brief preview of results

The research presented here combines theory and analysis of experimental data, which in turn include both choice behavior, information on personality, and brain imaging data. The path leading to results is long, so a preview of the results may be useful.

We first derive general properties of the optimal allocation of attention in a general theoretical setup where evidence is accumulated over time, with leakage. The driving force of the results is the trade-off between the need to spread attention effort over time (because of the convexity of the flow cost function) and the preference to concentrate effort in later periods, closer to the choice, because evidence decays with time. The balance of these two forces makes the optimal attention path increase at an exponential rate over time, with an exponent that combines the cost and the decay parameters.

Our analysis of experimental data shows that the decay effect, even for the short delay between the presentations of the two options, is significant and sizable, reducing, everything else being equal, the probability that the option presented first is eventually chosen (*recency* effect). The size of the recency effect is correlated with performance in working memory tasks of the participants. It is important to emphasize that the recency effect occurs only for specific values of the parameters. Our analysis makes this dependence of the effect on parameters clear; we do not claim that recency effect always occurs in sequential choice. Our model indicates conditions in which recency or primacy (both are typically found in judgment tasks, see for example [3]) may occur.

The model makes predictions on paths of brain activity during the task. Using *fMRI* data, we show that activity in the posterior parietal cortex, a brain region well known for its role in working memory, supports the memory system for values that we postulated in the analysis of the choice data. Thus, the use of brain imaging data and neural modeling is important in our analysis to give direct evidence supporting or rejecting the model.

## Materials and methods

### Experiment design

The design of the experiment is a special case of the general setup we will describe in our model. The study did not include minors. The participants in the study provided informed written consent to participate. The ethical committee at the University of Minnesota approved of the study (protocol number IRB 1002M78152).

We call option a pair (*x*, *d*) of an amount *x*, measured in dollars, and a temporal delay of the payment, denoted *d*, measured in days. We use the term *offer* as short for "presentation of an option". In each trial a participant could choose between an option offering a smaller reward delivered sooner, called early payment option, or *early* option for short, denoted (*x*_1_, *d*_1_); and an option offering a larger reward delivered later (late option), with (*x*_2_, *d*_2_); so *x*_1_<*x*_2_ and *d*_1_<*d*_2_. The early option was always colored in blue and the late option in yellow. Among the 54 early options, 25 had an immediate payment (*d*_1_ = 0, these are called *immediate-early*).

The two options were not presented simultaneously, but *sequentially*, for a total of 54 trials in two separate runs separated by a break. After the sequential presentation of the two options, participants could see a third screen in which the two options appeared together. At this point they could indicate their choice by pressing either a blue or yellow button on the response system, corresponding to the early (blue) or the late (yellow) option. The choice screen remained visible until the choice was made. After 6 seconds, if no choice had yet been made, a message *Choose Faster* (written in color red) appeared. There was a random time interval of 2 or 4 seconds between each screen presentation of the options, and between each trial. The entire inter-temporal choice task lasted on average 15 minutes.

We explained clearly to participants that the time sequence of presentation of the options would follow the pattern that we have just described. They were also informed that they would be paid the real amount at the appropriate time with a check sent by mail for one choice out of each of the two runs.

Our design is one of the many potential tests of a theory of choice with sequential presentation of options. It has some advantages and some shortcomings. The joint presentation of the two options before choice allows us to test the hypothesis that evaluation of options at earlier stages, and storing in working memory, occurs even when it is not strictly necessary for choice. Removing the joint presentation would make the intervention of working memory in the process mandatory and thus obvious. Our design and results highlight that the storage of value in working memory is an essential feature of choice: automatic processes are in place that induce storage of value and choice in real time, as soon as the relevant information is available. The risk in our setup was that the joint offer might have made the earlier sequential presentations irrelevant, because participants might have ignored them. As we are going to see, this did not happen.

As we mentioned, a warning message inviting to choose faster was displayed after a time interval. Some way of enforcing a time limit is necessary in an experimental test, and the message displayed is a mild way of implementing it. The time interval before the warning is so long (6 *s*) that the constraint is in fact inactive in the experiment. As we discuss more in detail in the section analyzing the response time (*RT*), the mean *RT* is 830.3 *ms*, with a *SD* = 636.3 *ms*, approximately log-normally distributed, thus largely below the time constraint which is necessary in any experiment.

In the group of participants (*N* = 96) that we consider here, the presentation of the two types of options is randomized. For completeness, we mention here that, for a different subgroup (*N* = 152) of the entire sample in the study, the order of presentation of the two options was fixed: participants always saw the early options first, and the late option second. This alternative procedure is consistent with designs used previously. For instance, in [4] participants had to choose between an option presented on the screen and a default option. The latter was always the same: an immediate payment of $20. The design in [5] is similar (the default, immediate payment, option was $10). For these participants the moment of the choice occurs at the presentation of the late option, and they differ from the ordered presentation described here because in our task the first, early payment option was different in every trial. We do not use this part of the sample in the current study because of the confound between early payment and presentation as first option. For the record however behavioral analysis in this sub-sample also shows the recency effect that we document here for the sub-sample being considered, and it is approximately of the same size.

Participants always knew which type of option was presented at each moment, given the color code of the early (blue) and late (yellow) options.

### A model of memory and attention

In this section we consider a general model of attention and memory for choices made when options are presented sequentially. The setup is general, and includes several possible experimental designs, in particular the one we just presented. We consider a necessary requirement for a theory of attention and memory that predictions should be offered for a variety of experimental setups, and the predictions should derive in all cases from the same general principles. Special interesting instances included in the theory are the case of simultaneous presentation and the case of the sequential presentation, considered later.

The analysis will highlight the role played by a basic trade-off between cost of attention and decay of evidence, and it may be useful to describe it to offer guidance. Since evidence decays, the decision maker might be inclined to concentrate the attention and thus the information gathering in a short time period, closer to the moment of choice, so that the evidence gathered does not suffer from decay. Since the cost function is convex in effort, on the other hand, it might be better instead to spread the attention over longer periods of time, not to incur steeper costs induced by the convexity at high levels of attention effort. The optimal allocation over time results from a delicate balance between these two opposing forces, summarized by the result that the attention provided is increasing over time at an exponential rate which is equal (see Eq (21)) to the ratio of a parameter determining how fast evidence decays and a parameter determining the cost of attention. This conclusion does not depend on the specific form of the sequential presentation and is thus a general feature of all the choice environments with sequential availability of options.

We now define the optimization problem, first introducing the necessary prerequisites. We first define utility and attention costs, then the stochastic process underlying the flow of information, then the timing of choice, and finally we define the problem of allocation of attention with decay of evidence. The reader who is not interested in the analytical details of the model can skip from here to the Results section.

### Utility and attention

A *DM* has to choose one option out of a menu of two, denoted *A*, *B*. The utility of each option is determined by a state of nature, chosen in a finite set of states, denoted by *Θ*. The true state *θ* is unknown to the *DM*. There is a probability *π* on *Θ* determining the frequency of *θ*. The utility of option *o* at state *θ* is denoted *u*(*θ*, *o*). An option *B* is dominated by option *A* if the utility from *A* at all states is larger than that from *B*, and strictly larger in at least one state. We assume that there are no irrelevant states, that is for all *θ*, *π*(*θ*)>0. The *DM* can accumulate over time evidence in favor of each of the two options, deciding to pay more or less attention to the information available on the utility of the option. This accumulation process takes place in a time interval [0, *t*_*F*_], where *t*_*F*_ denotes the final time: a choice has to be made before this limit, or the option is lost. The two options are available for inspection at given sub-intervals, possibly together. So the total interval [0, *t*_*F*_] is partitioned into subsets of the form *I*_*k*_ for *k*∈∅, *A*, *B*, *A*, *B*, where the index *k* indicates the set of options which are displayed. Choice can be made within a sub-interval of [0, *t*_*F*_], the choice interval.

We now consider the optimal allocation of attention. At any point in time *s*∈[0, *t*_*F*_] the *DM* can allocate a level *a*(*s*,*o*) depending on the time *s* and the option *o* in a given set of feasible attention efforts which we now describe. When the option is not available for inspection, then effort in observation of that option is not feasible (or, equivalently, has no informative value, or has infinite cost). When only one option is available at time *s*, attention can be devoted to it, at any level in a given interval *F*(*s*) of the reals, where:

$$F(s)\equiv [\underline{a}(s),a^{-}(s)]$$ (1)

The time independent constraints (which will be indicated simply by *a* and ^−^), and the unconstrained cases (with *a* = 0 and/or *a*^−^ = +∞) are allowed. We will state explicitly when the conditions *a*>0 or *a*^−^<+∞ are introduced (in particular the possibility *a*>0 is discussed below, when we formulate predictions on behavior in our experimental setup.).

When both options are available, attention can be allocated to both options at the same time, but then the sum of the two attention efforts is constrained to be in *F*.

In summary, attention has to satisfy the following two feasibility constraints:

$$a(s,o)=0\;if\;s\notin I_{o}\cup I_{A,B}$$ (2)

∀s:a(s,A)+a(s,B)∈F(s). (3)

The possibility that a minimum strictly positive attention is devoted to a displayed option is suggested by an established regularity in research on reward evaluation in animal (including human) studies. For example, in single neuron recording studies activity associated with evaluation ramps up starting immediately after the display of the reward, reaches a peak and then declines to baseline within 1*s* of the display. This is equally true for studies based on a Pavlovian design as it is for choice designs. In a Pavlovian design, a reward is presented or announced by a cue, and no choice is possible (the cue only tells that a reward is forthcoming); in a choice design, two options are presented and the participant can choose between the two. The time profile of neural activity is the same in both cases. This is well known: see the pioneering [6] study (which adopts a Pavlovian design: see also [7]) and [8] (which instead adopts a choice design; see [9] for a model predicting this time path) shows this clearly.

In our design, participants were informed that they could choose at any time after the onset of the joint presentation of the two options. We take a very cautious position and interpret the design in our model as saying that no choice was possible after the warning shown after 6*s*, and take the onset of the warning message as the final time *t*_*F*_. Given the condition described in Eq (2), the attention allocation when no option is presented is trivial and we ignore it in the analysis. We have 0<*t*_1_<*t*_2_<*t*_*F*_ and the following intervals:

$$I_{A}=[0,t_{1});I_{B}=[t_{1},t_{2});I_{A,B}=[t_{2},t_{F}].$$ (4)

The choice interval is [*t*_2_, *t*_*F*_] The length of the time intervals between the presentation of the two isolated options are random; the length of time *t*_2_−*t*_*F*_ is 6 *s*. After *t*_*F*_, a random time inter-trial time interval followed, and a new trial started.

### Informative signals

In our model the decision maker does not have a belief on the state that he updates on the basis of available evidence provided by a stochastic process: the probability *π* is objective. Thus, our analysis differs from existing research within the Bayesian framework, in which the decision maker updates a belief on utility relevant states. We assume that attention is chosen optimally, and so the rate of evidence accumulation is endogenously and optimally determined depending on the environment; in this respect our approach differs from Drift Diffusion Model (*DDM*). Our approach is closer to recent work on attentional Drift Diffusion Model (*aDDM*), ([10], see [11] for a very clear recent exposition). It differs from these contributions in an important respect, namely that the evidence accumulated decays over time.

Informative signals are taken to be a stochastic drift diffusion process. We denote *W* the standard Brownian motion (zero drift and unit variance) and build the signal process on it. The signal process *X*(*t*)_*t*≥0_ depends on the vector of state of nature, attention and option, (*θ*, *a*, *o*); it is a drift diffusion process with constant variance *σ*^2^, and a drift *γ*(*θ*, *a*, *o*).

The accumulated evidence associated with each option proceed separately and independently, until the process is stopped and a choice of one of the two options is made, favoring the option that has the highest favorable evidence. The *DM* regulates the attention over time. We first define precisely the feasible set of choices.

**Definition 1** A path is a measurable function from time to attention effort; the set *A* of feasible attention paths is the set of paths that satisfy the feasibility constraints (2) and (3).

We emphasize that a choice of attention is a time path, and not a policy; thus for example the stopping time at which choice is made is decided ex-ante. This is consistent with the nature of the investigation here, aimed at identifying stable patterns of attention on the basis of biological basis, rather than analyzing what the optimal choice would be, for example by a Bayesian learner. Given an attention path a, we call *T*(*a*) the time at which the participant chooses. This stopping time does not depend on the evidence accumulated, as instead does in the *DDM*. Given the setup developed in detail later, it is natural to assume that he decides as soon as he stops acquiring evidence, because with no effort the available information can only deteriorate. So we define:

$$T(a)\equiv inf\{s\in [0,t_{F}]:\underset{o}{max}\{es{sup}_{t\in [s,t_{F}]}a(t,o)\}=0\}$$ (5)

and we will assume that the decision maker communicates the decision in the first time in the admissible set that follows *T*(*a*). In the following analysis we may sometimes drop the *a* in *T*(*a*), when no ambiguity can arise.

What we have introduced is a version of a race model between the two options. Taking the difference between the two accumulated evidence we have a version which is related to the *DDM*, in the more general version that includes attention (the *aDDM*). Differently from the *DDM*’s, the time at which the information gathering stops is endogenously determined in our model, and is given by the time at which the attention effort is set to zero. By the properties of the model (namely that noise accumulates even when no effort is provided) it is optimal to choose on the basis of the available evidence when effort stops.

### Information decay

We now introduce the crucial element, the decay of the evidence accumulated. This occurs in two distinct ways, examined below. The two processes, one that gathers evidence and one that stores it, are qualitatively different and located in different brain regions: reward evaluation areas are relevant for the first, working memory areas for the second. So it is important to introduce them separately.

### Signal decay

For a given stochastic process *X*(*t*)_*t*≥0_ and a real-valued leak parameter *ρ*≥0, the *leaked process* (representing the evidence currently available) is defined by:

$$dLX(t)=-\rho LX(t)dt+dX(t),t\geq t_{0};X(t_{0})=x_{0}\;a.s.$$ (6)

When *X*(*t*)_*t*≥0_ is a drift diffusion process with constant coefficients then Eq (6) is the Langevin equation and the leaky process is the Ornstein-Uhlenbeck (*OU*) process. The following proposition describes the leaked process explicitly.

**Proposition 2** *The process defined by the stochastic integral*:

$$LX(t)=\int_{0}^{t}e^{-\rho (t-s)}dX(s)+e^{\rho t}x_{0}$$ (7)

is a solution of the differential Eq (6).

The statement follows from Ito’s formula.

### Memory decay

When no information on the value of an option is gathered, the current value can be stored in memory, and follows a process of the same form as described in Eq (6), with two differences.

First, the leak parameter may be different. We will temporarily use *ρ*_*M*_ to indicate this possibly different value. Second, in this case the underlying process *X*(*t*)_*t*≥0_ is independent of state, action or effort. As we indicate below, allowing the difference does not change the qualitative substance of the results. In the case of the drift diffusion process, if we set the drift equal to zero, we have:

$$dLX(t;\theta ,A)=-\rho_{M}LX(t;\theta ,A)dt+\sigma dW(t).$$ (8)

The general model where *ρ* and*ρ*_*M*_ are different can be analyzed along the lines we follow in the next sections, but with considerable complications in the notation and little gain in insight and generality. In conclusion, we assume:

$$\rho =\rho_{M}$$ (9)

because the gain in simplicity more than compensates the small loss in generality.

### Optimal attention allocation

We now study the main properties of the optimal allocation of attention.

### Cost of attention

The flow cost of the attention effort is a function *c* of the sum of the attention efforts devoted to the options; this flow cost is not discounted, so the total cost from an attention path a is:

$$C(a)\equiv \int_{0}^{t_{F}}c(\sum_{o}a(s,o))ds.$$ (10)

Our analysis only requires the running cost function to be convex. To make the analysis more transparent we choose a power form for the cost:

**Assumption 3** *The attention cost function is*:

$$c(a)=a^{R},R\in (1,+\infty ).$$

We will occasionally use the notation *c*, and *c*′ for the derivative, to make the exposition less cumbersome.

### Utility

We have modeled, as usual in decision theory, the uncertainty of the decision maker by introducing a variable *θ* which describes a state of nature unknown to the agent performing the maximization. For a given path of attention effort a and a probability *π*∈*Δ*(*Θ*), we define the expected utility over the unknown state *θ* as:

$$E_{a,\pi }U\equiv \sum_{\theta ,o}\pi (\theta )u(\theta ,o)P(\left\{LX(T;\theta ,a(\cdot ,o),o)>LX(T;\theta ,a(\cdot ,o^{\prime }),o^{\prime })\right\})$$ (11)

where *o*′ denotes the option in the menu different from *o*.

### Optimal allocation

We have now all the elements to define the optimization problem.

**Definition 4** Given a probability *π*, the optimal attention allocation problem is the maximization of the expected utility (as defined in Eq (11)) minus the cost (defined in Eq (10)) over feasible attention paths (as in definition 1); so the objective function is:

$$\underset{a\in A}{max}\;(E_{a,\pi }U-C(a))$$ (12)

In interpreting the objective function (12) we do not commit to a specific identification of who performs the maximization. In particular we do not assume that this maximization is a conscious choice of the decision maker. Different interpretations are possible: for example the choice of the policy is the outcome of learning, or of an evolutionary process selecting an adaptation to the environment.

We now show that the optimization problem (12) can be rewritten in a simple form, as in (15) below. Define first the total weighted attention devoted to the option *o* up to time *t*, given a pair a=(a(⋅,A),a(⋅,B)), as:

$$G(t,o;a)\equiv \int_{0}^{t}e^{-\rho (t-s)}a(s,o)ds$$ (13)

We rewrite the maximization policy to highlight the property that only the pair of the two weighted attention efforts defined in (13), and the stopping time *T* matters. Recall *W* is the standard Brownian motion; we define the weighted noise process for option *o* as [12]:

$$W_{\rho }^{o}(t)\equiv \sigma \int_{0}^{t}e^{-\rho (t-s)}dW(s)$$ (14)

This is the noise accumulating on the signal for option *o*, modeling the exogenous random disturbances, distractions, stimuli extraneous to the choice process and so on, that begin to accumulate starting at initial time. The following proposition is now clear:

**Proposition 5** *For every attention path*:

$$P(\left\{LX(T;\theta ,a(\cdot ,o),o)>LX(T;\theta ,a(\cdot ,o^{\prime }),o^{\prime })\right\})=P(\left\{G(T,o;a)u(\theta ,o)-G(T,o^{\prime };a)u(\theta ,o^{\prime })>W_{\rho }^{o^{\prime }}(T)-W_{\rho }^{o}(T)\right\})$$ (15)

The random variable $W_{\rho }^{o^{\prime }}(T)-W_{\rho }^{o}(T)$ is normal, zero-mean, and only depends on T and ρ through its variance.

The variance of the random variable is well known, and we recall its properties in lemma 10. Therefore the expected utility (without considering cost of information) only depends on the triple (*T*, *G*, *π*), where $G\equiv (G(T,o;a{)}_{o\in A,B})$, and we write it as *E*_*T*,*G*,*π*_*U*.

### Constant returns to effort

We now introduce an assumption on returns to effort which needs some introductory motivation. The potential problem is that of the chattering control, that is an optimal control (attention in our case) that over time oscillates between two values to convexify the resulting value. To illustrate the potential problem, we consider a simple example. Let *ρ* = 0 (a positive leak is not necessary for this illustration, because this potential difficulty has nothing to do with the leak), so that the outcome of the attention effort results from the accumulated, unweighted integral of the effort. Let the drift of the signal process be *γ*(*θ*, *a*, *o*) = *a*^*α*^*u*(*θ*, *o*), where *α*≤1, and cost function *c*(*a*) = *a*^*R*^, we can define a monotonically increasing transform of *a* as *a*^~^≡*a*^*α*^. The drift with the new variable at (*θ*, *o*) is *a*^~^*u*(*θ*,*o*) (so, linear in attention effort) and the *cost* is ${a˜}^{\frac{R}{\alpha }}$. If $\frac{R}{\alpha }<1$ then the cost is a concave function of the new effort variable, and so the optimal policy is to chatter, that is, switch from high to low levels of attention as quickly as possible.

Our re-definition of the effort variable to get a linear effect of the attention variable on the drift takes the following general form requiring the to be homogeneous of degree one in effort:

**Assumption 6** For all (*θ*, *a*, *o*), *γ*(*θ*, *a*, *o*) = *aγ*(*θ*, 1, *o*).

For the record, the corresponding assumption needed in the case of a Lévy process is that for all (*θ*, *a*, *o*):

(γ(θ,a,o),C(θ,a,o),ν(θ,a,o))=a(γ(θ,1,o),C(θ,1,o),ν(θ,1,o))

In words, we are requiring that there is a scaling of the effort variable such that in that variable the drift is linear and the cost function convex. With this scaling of the effort variable, chattering is not possible.

### Cost minimization

For some purposes it is convenient to reformulate the optimization problem as a two-step problem, in which first *(T*, ***G****)* is chosen, and then the cost of achieving ***G*** in time *T* is minimized:

$$\underset{a\in A}{max}\;(E_{a,\pi }U-C(a))=\underset{T,G}{max}\;(E_{T,G,\pi }U-\underset{a:G(T,\cdot ,a)=G}{min}C(a));$$ (16)

Eq (16) implies that a path a that maximizes expected utility net of cost also solves the cost minimization problem among the effort paths that have the same induced *T* and ***G***. We will use this property to characterize the optimal effort path below.

We now study the properties of the indirect cost function, assigning the minimum cost to yield a pair ***G*** of total weighted attention efforts within time:

$$\underset{a:G(T,,:a)=G}{min}C(a)$$

### Simultaneous display

We begin with the most common experimental setup, in which both options are simultaneously displayed, that is the case in which there is a single component in the partition introduced in our general setup:

$$I_{A,B}=[0,t_{F}]$$ (17)

and decision can be taken in any time interval [*t*_2_, *t*_*F*_], for some *t*_2_<*t*_*F*_. For any fixed time *T*, and a given pair of total attention efforts that we write (dropping the pair (*T*, a) to lighten notation) (*G*(*A*), *G*(*B*)), we solve:

$$\underset{a\in A}{min}C(a),subject\;to:$$ (18)

$$\int_{0}^{T}e^{-\rho (T-s)}a(s,o)ds\geq G(o),o\in A,B.$$ (19)

The solutions of the problem are characterized in proposition (7), which shows some of the important features of the optimal allocation of attention when evidence decays over time. The fist feature is the increase in attention over time to counter the effect of decay, as we had announced at the beginning of this section. Depending on the parameters (for example, on how long is the interval in which options are available for inspection) no attention might be paid in the early stages; when attention is given, then it is increasing, with marginal cost increasing exponentially over time (that is, with effort increasing over time). More precisely:

**Proposition 7** Assume both options are displayed at all times (i.e (17) holds). The problem defined by (18) and (19) has a non-empty set of solutions. There is an *s*∈[0, T) such that

1. *For all s*∈[0, *s*), ∑_*o*_*a*(*s*, *o*) = 0;
2. *in the interval* (*s*, *T*], ∑_*o*_*a*(⋅,*o*) *is strictly positive*, *increasing;*
3. *for all s*∈*(**s*, *T*]:

$$c^{\prime }(\underset{o}{\sum }a(s,o))=\lambda e^{-\rho (T-s)}$$ (20)

for a positive constant λ.

It is clear that if the derivative of the cost function is zero at zero effort (as is the case under assumption (3)) then *s* = 0. Also it is clear from the proof (see comments following equation (S-7)) that some attention is given to at least one of the options unless the problem is the trivial one in which in all the states that have positive probability, the two options give the same utility. Given the functional form of the cost function, the proposition has a simple implication which illustrates a general feature of the optimal allocation of attention. Using assumption (3) in Eq (20) a simple computation shows that the total attention in the interval (*s*, *T*] is an exponential function of time, at a rate which is a simple combination of the leak rate and the power of the cost function. This rate will appear frequently, so we give it a special notation:

$$\rho_{R}\equiv \frac{\rho }{R-1}$$ (21)

The exponential increase over time is an important general property of the optimal effort provision: everything else being equal, effort is preferentially provided at later times, because the evidence gathered earlier is subject to decay. Note that a higher leak parameter induces a relatively higher postponement, and a higher power of the cost function has the opposite effect.

### Sequential display

We now consider the general case with sequential display. The objective function (18) is unchanged, but the constraint (19) is replaced by:

$$\int {}_{\left(I_{o}\cup I_{A,B})\cap [0,T\right]}e^{-\rho (T-s)}a(s,o)ds\geq G(o),o\in \{A,B\}.$$ (22)

As in proposition (7) we have that effort can only increase over time, and that when effort is positive at two different points, then the marginal cost at these two points is proportional to the exponential of the time difference, multiplied by the leak factor *ρ* (as, for example, in equation (S-21) below). It may be useful to keep in mind the quadratic cost function, in which case, for example, that equation links attention in two different periods as $a(t,A)=a(s,A)e^{\rho (t-s)}$.

We characterize the effort paths when *I*_*A*,*B*_ follows *I*_*A*_ and *I*_*B*_. For simplicity we set *I*_∅_ = ∅ (so $t_{i}^{\prime }=t_{i}$ for = 1,2), since the attention is restricted to 0 when no option is displayed. This is the case which is relevant for the analysis of the data in our experiment, where the joint presentation of the two options is last. With this case in mind we define the first time the attention to an option becomes strictly positive:

$${\underline{s}}_{o}\equiv sup\{s:es{sup}_{t\in [0,s]}a(t,o)=0\}$$ (23)

**Proposition 8** Assume that *I*_*A*,*B*_ follows *I*_*A*_ and *I*_*B*_; then for all *o*:

$$1\;\forall r\geq s>s_{o},r,s\in I_{o}$$

$$c^{\prime }(a(r,o))=c^{\prime }(a(s,o))e^{\rho (r-s)}$$

$$2\;\forall r\geq s>s_{o},s\in I_{o},r\in I_{A,B}$$

$$c^{\prime }(\underset{b}{\sum }a(r,b))\geq c^{\prime }(a(s,o))e^{\rho (r-s)},$$

*and if the inequality is strict*, *then a*(*r*, *o*) = 0.

We illustrate the proposition in the case of the quadratic cost function. Once the attention devoted to an option *o* in *I*_*o*_ becomes positive, it stays positive afterwards, and the marginal cost grows in time at the exponential rate *ρ*; the marginal cost of total attention in the interval *I*_*A*,*B*_ also grows at that rate. If the baseline of the total level of the attention in *I*_*A*,*B*_ is larger than that of *o*, than no attention at all is paid to *o* in *I*_*A*,*B*_

Many cases are possible, depending on the parameters of the problem, *u*, *π*, and *t*_1_, *t*_2_, *t*_*F*_. For example, if *t*_1_ is substantially shorter than *t*_2_−*t*_1_, and *t*_*F*_ is close to *t*_2_, then attention is devoted to the first option in the first interval ([0, *t*_1_]) and the last ([*t*_2_, *t*_*F*_]), and no attention is paid to the option presented second in the joint display interval. In proposition 9 below we will see more precise conclusions in the special case of our experimental design.

### Model predictions for the experiment

We now consider the optimal allocation of attention and memory in the case of our experimental design, with the special structure of the display intervals as specified in Eq (4). At *t*_1_, or at any earlier point in which the attention effort is reduced to zero, the accumulated evidence is transferred to a working memory network, where it may decay; this process is described by Eq (8).

As the second option is presented, attention is devoted to it in the interval between *t*_1_ and *t*_2_. At time *t*_2_, both options are displayed, and attention may be devoted to both options. The value *LX*(*t*_2_; *θ*, *a*(⋅,*A*), *A*) is retrieved from working memory, while the value *LX*(*t*_2_; *θ*, *a*(⋅,*B*), *B*) is given by the process of cumulating evidence until *t*_2_; these are the two new initial conditions for the process described by the Eq (6). An effort *a*(*s*, *o*)∈*A* for *s*≥*t*_2_, an option *o*∈*A*, *B* is chosen, until the process is stopped or the final time *t*_*F*_ is reached and a choice is made; the option with highest value of accumulated evidence at that point is chosen. Having specified a precise setup in the sequential presentation allows us now to tighter predictions.

The main prediction follows from the analysis of the implications of the *steepness of the cost function at zero*. We call a cost function flat or steep depending on whether the exponent is larger or smaller than 2. A flatter cost function makes the incentive to spread the allocation over time stronger. On the contrary, a steeper cost function gives the opposite incentive to concentrate attention in more restricted time and decide as soon as possible. In our experimental setup, a steep cost function will make it optimal to gather information in the first presentation, and the evidence on the option presented first will be more harmed by the decay, and thus that option will be less likely to be chosen. In summary, we should expect that a choice made immediately (as soon as choice is feasible) should go together with a recency effect and be due ultimately to a steeper cost function. This is what we see in behavioral data.

We first consider the optimal policy in our experimental setup.

### Optimal policy in the experiment

The next proposition characterizes the optimal allocation in the case we are considering:

**Proposition 9** The optimal attention allocation has the form, for some vector of non-negative real numbers (*x*_1_, *y*_2_, *x*_2_, *y*_2_):

$$a(s,A)=x_{1}e^{\rho_{R}s},s\in [0,t_{1})$$ (24)

$$a(s,B)=y_{1}e^{\rho_{R}(s-t_{1})},s\in [t_{1},t_{2})$$ (25)

$$\underset{o}{\sum }a(s,o)=(x_{2}+y_{2})e^{\rho_{R}(s-t_{2})},s\in [t_{2},T].$$ (26)

The proof makes clear that at least one of *x*_1_ and *y*_1_ strictly positive, and thus some attention is given, unless for all *θ*, *u*(*θ*, *A*) = *u*(*θ*, *B*). In this latter case paying no attention and choosing with arbitrary probability is obviously optimal. We emphasize for future reference that the statements holds also when *ρ* = 0; in this case the proposition states that attention is constant over time. We rely on this proposition to establish the link between the time in which the decision is made, and the recency effect developed in the next section.

### Effort cost and recency effect

The form of the optimal attention described in proposition (9) is independent of the value of the power of the cost function, *R*. Two important features instead depends critically on this value, and in particular on whether *R* is larger or smaller than 2: whether a decision is taken as soon as allowed, and whether there is a recency effect. We will see that these two features are linked: recency effect is associated with immediate decision, and this depends on whether *R*<2 or not.

It is important before we continue to note that an essential feature of the problem we are considering, namely the fact that the noise in information grows with time. Our model has no discount: however, a *ρ* positive makes, everything else being equal, an earlier decision better, because information is decaying. Thus, decay of information has an effect like discount. There is however an additional incentive to reach an earlier decision, which holds whether *ρ* is positive or not, and it is provided by the variance induced by the noise term. To see this, we consider the variance of $W_{\rho }^{A}(T)-W_{\rho }^{B}(T)$, the total noise, which is independent of the attention level (the noise terms $\left(W_{\rho }^{A}(T):o\in A,B\right)$ were introduced in Eq [14]. The next lemma show the additional incentive to choose earlier, independently of the value of:

Lemma 10 The function $T\to Va(W_{\rho }^{A}(T)-W_{\rho }^{B}(T))$ is:

$$\sigma^{2}(\rho ,T)\equiv \frac{\sigma^{2}}{\rho }(1-e^{-2\rho T})$$ (27)

with *σ*^2^(0, *T*) = 2*σ*^2^*T*, thus increasing in *T* for any *ρ*≥0.

Therefore the noise in information grows with time for all values of *ρ* including 0. This fact makes, everything else being equal, an earlier decision better, even when the leak parameter is zero. Of course, the clause "everything else being equal" is important: as *T* grows the difference between *G*(*T*, *A*) and *G*(*T*, *B*) also grows (see, for a related result, example 3 of [13] and Fig 1A of [14]).

**Fig 1:**
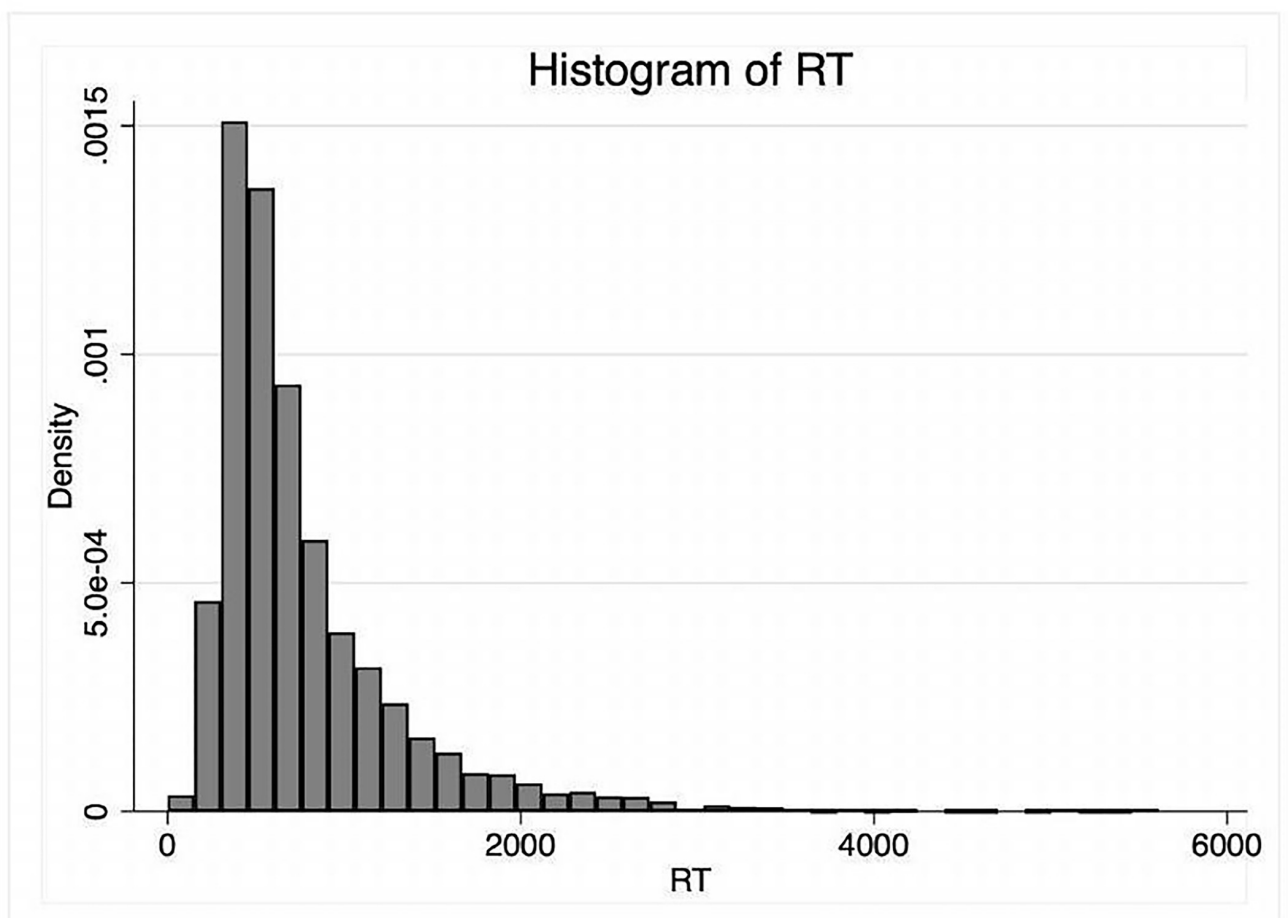
Distribution of RT. Raw response time reported here; the horizontal scale is in *ms*. For a log-transformation.

We can now discuss the role of the power *R*. To offer an intuitive reason for the results, we note an obvious but crucial difference between the case *R*>2 or not: although in both cases the marginal cost tends to zero as the effort tends to zero, the derivative of the marginal cost, *c*″(*a*) = *R*(*R*−1)*a*^*R*−2^, tends to zero as effort tends to zero if *R*>2, and instead tends to infinity if *R*<2. Our data indicate that *R*<2.

We rely in the following analysis on the feature of the experimental design that the amount of time available for inspection of the two options in the single option display is the same, that is:

$$t_{1}=t_{2}-t_{1}$$ (28)

Recall that in our design the allocation of the two rewards is balanced, so if a pair of rewards is presented as (*A*, *B*) the same pair is presented as (*B*, *A*). To study the conditions that produce a recency effect we consider the case in which the utility of the two options is the ex ante the same. In this case the only difference between the two options is in the order and length of the time intervals in which the options are presented, so the potential order effect on choice, for example recency, can be completely separated from the effect of different utilities.

So we will assume in the rest of this subsection the simplifying assumption of *N* even, the *π* is uniform:

$$\pi (\theta^{i})=1/N$$ (29)

and the utility is symmetric:

$$u(\theta^{i},A)=u(\theta^{N-i+1},B)=1,i<\frac{N}{2},zu(\theta^{i},A)=u(\theta^{N-i+1},B)=0,i>N/2.$$ (30)

To make the discussion more precise, we write *V*(*a*, *T*) the functional to be maximized on feasible (*a*, *T*) pairs of attention effort paths and stopping time *T*.

The next step is particularly easy to understand in the special case in which there is no leak; so until further notice we assume that *ρ* = 0. In this case, when utility, probability and presentation time length are symmetric, proposition 9 gives that the optimal effort is a constant value equal for both options. The total effort devoted to either option is the same that is:

$$E(T,A)\equiv \int_{0}^{T}a(s,A)=E(T,B)$$

This common value will be denoted by *E*(*a*, *T*). We let *V*(*a*, *T*) denote the function of the attention level and decision time, in the case = 0:

$$V(a,T)=2\Phi \left(\frac{E(a,T)}{\sigma (0,T)}\right)-c(a)T$$ (31)

with *E*(*a*, *T*) = *aT* and $\sigma (0,T)=\sqrt{2}\sigma T^{1/2}$. The following shows the reason why the optimal solution has different features on the two sides of the value *R* = 2. We define as usual the sign function as 1 at positive values, -1 at negative values, and 0 at 0.

**Proposition 11** Let *ρ* = 0. On (0,*a*^−^]×[*t*_2_, *t*_*F*_]: if *R* = 2 then *V*_*a*_(*a*, *T*) and *V*_*T*_(*a*, *T*) have the same sign; if *R*<2 then *V*_*T*_(*a*, *T*)≥0 implies *V*_*a*_(*a*, *T*)>0; and if *R*>2, then *V*_*a*_(*a*, *T*)≥0 implies *V*_*T*_(*a*, *T*)>0.

### Boundary property

From the proposition we conclude an important feature of the dependence of optimal solution on the value of: except in the case *R* = 2, one of the two variables is on the boundary of the feasible choices, and which one it is depends on whether *R*<2 or not. For example, when *R*<2, the derivative of the marginal cost increases at zero, and thus the optimal attention stays away from zero, and is in fact at the upper boundary.

The following corollary gives the precise statement. It states that the optimal solution has a boundary property in the attention-time space, of a very specific nature depending on the value of the exponent *R*. When *R*<2, the optimal choice is either to decide as soon as possible or to use maximum attention. When *R*>2, the optimal choice is either to decide as late as possible or to use minimum attention.

**Corollary 12** Let *ρ* = 0, and consider the feasible region [*a*, *a*^−^]×[*t*_2_, *t*_*F*_], a rectangular region in $R_{+}^{2}$. The optimal solution (*a*^^^, *T*^^^) has the following properties:

1. *It is always (that is*, *for all values of the parameters) on the boundary of the region*, *except when R* = 2;
2. *When R*<2 *it is always on the east* (*T*^^^ = *t*_2_) *or north* (*a*^^^ = *a*^−^) *sides;*
3. *When R*>2 *it is always on the west* (*T*^^^ = *t*_*F*_) *or south* (*a*^^^ = *a*) *sides;*

### Optimal attention and recency bias

We now return to the general case in which *ρ*≥0. The evidence offered by the analysis of behavioral data (in particular that of the response time) suggests that the cost function of attention effort is closer to a power function with exponent less than 2, since the decision is taken almost immediately after *t*_2_. In these case, for small but positive values of *ρ*, recency bias follows. More precisely, the conclusion in proposition 13 below holds.

Consider the benchmark case in which the two options are in all respects identical, except for the fact that *A* is presented first; so assume (29) and (30). We call the values *x*_1_ and *y*_1_ in proposition 9 the attention values: the attention paid to an option is a path, but as stated in the proposition, the path has the very specific exponential form, so it is completely characterized by the initial value (respectively *x*_1_ and *y*_1_).

In the proposition we focus on the case which is relevant on the basis of the behavioral data that show that choice is taken in very close proximity of the first moment in which choice is allowed. From our analysis in the case *ρ* = 0 we know that this is the optimal choice of time when the parameter *σ* is sufficiently large, or the length of time in which the two options are presented is long enough. In this case it is optimal to start collecting information immediately because the noise is accumulating from the initial time.

**Proposition 13** Consider all the values of the parameters for which the optimal choice of time *T* = *t*_2_, and either the optimal path is interior for both options, that is

$$\forall o,\forall s\in I_{o},a(s,o)\in (\underline{a},a‾)$$

or entirely constrained, that is:

$$\forall o,\forall s\in I_{o},a(s,o)=\underline{a},$$

or

$$\forall o,\forall s\in I_{o},a(s,o)=a‾$$

then recency bias occurs, that is, the probability of the option presented second is higher.

An intuitive understanding of the result can be obtained in two steps. First consider the case in which *a*>0, and is large enough so that the constraint is binding over the entire interval in which the option is presented. In this case by necessity an attention *a* is allocated to both options. But since the attention allocated to the option presented second decays at the rate *ρ* over a smaller time horizon, the probability of the second option being chosen is larger. The situation is similar when the upper constraint *a*(*s*, *o*)≤*a*^−^ is binding over the entire presentation interval. As a second step, consider the case in which at *ρ* = 0 there is an interior solution, call it a*, common to both options. In this case from proposition 9 we know that the optimal paths for *a*(⋅,*A*) and *a*(⋅,*B*) have the exponential form described in Eqs (24) and (25). Thus, the paths are entirely characterized by the initial values *x*_1_ and *y*_1_. By our assumption that *T* = *t*_2_ there is no attention allocated jointly. An easy computation (see the proof of the proposition in the Appendix) shows that the probability of option *B* being chosen more frequently is that at the optimal values *x*^^^_1_ and *y*^^^_1_:

y^1eρt1>x^1, (32)

which is indeed the case at the optimal choice. The reason why the inequality (32) is equivalent to recency bias is of course that the probability of choice is determined by the attention discounted by the leak, which takes into account the larger leak for the value accumulated for option *A*.

## Results

### Behavioral results

We first summarize the predictions of our model in our experimental environment. We focus here on predictions on choice that are specific to the model; other natural predictions (such as the association of the intelligence with the preference for delayed but larger payments, or a larger response time associated with closer values of the two options) will be reported as a check for consistency with established results.

1. In the environments of our experiment (as described in Eq (4)) we expect a recency bias.
2. The performance of the individual participant in the working memory task is decreasing in the leak parameter *ρ*, whereas the size of the recency effect in the choice task is increasing in *ρ*; thus the two should be associated (higher bias associated with lower performance in the working memory task).

The data in turn will allow us to provide some estimate of the two critical parameters of the model, namely the leak and the cost function. As we mentioned earlier it seems likely that (if the cost function is a power) that the exponent is less than 2. From our analysis of choice data the *ρ* parameter is approximately 7.4 per cent. The data needed for the analysis performed in the paper are available at DATAVERSE https://dataverse.harvard.edu/ at the following address: https://dataverse.harvard.edu/dataset.xhtml?persistentId=doi:10.7910/DVN/WTHVN8.

### Analysis of choice behavior

Analysis of our data using different functional forms for the utility of an option (for example, exponential discounting) shows that the estimate of the effect of memory does not depend on the specific functional form chosen. It is well known from 14 that the assumption of a linear utility of money can bias the estimate of the discount factor; thus, utility function and discount factor should be estimated together. For the next estimates, we take a Taylor expansion at (0,0) up to the second order and estimate the logit model taking the probability of the late option to be a logistic function of the difference in utility of the late and early option. For the hyperbolic utility the approximation we are using is therefore equal to *x*(1−*rt*), with *r*>0. It is easy to check that the adoption of exponential discounting would yield the same approximation. The analysis of Table 2 (where we need an estimate of the subjective value of the two options) relies on the hyperbolic discounting model:

$$V(x,t)=\frac{x}{1+kt}$$ (33)

with *k*≥0, consistently with the analysis already developed in [15].

Table 1 reports the panel data probit analysis of the choice of the Late option. The use of probit is preferable in view of the stochastic choice model we have presented (as opposed to that implied by the Drift Diffusion setup, for instance, which leads to a logit). The dependent variable is the choice of the Late payment option. The variables *am*. *late* and *am*. *early* indicate the dollar amount of the late and early payment option respectively; *time late* and *time early* the time delays in the payment of the two options. *IQ* is the Intelligence score (see section *S*−1.4 for a description of the task).

**Table 1:** Choice of the late option: Panel data probit. Standard error, clustered at the subject level, in parenthesis. Dependent variable: Choice of the late payment.

	(1)	(2)	(3)	(4)
	b/se	b/se	b/se	b/se
Am. late—am. Early	0.087***	0.074***	0.074***	0.072***
	(0.014)	(0.014)	(0.014)	(0.014)
Time late—time early	–0.079***	–0.084***	–0.085***	–0.077***
	(0.009)	(0.009)	(0.009)	(0.009)
Am. *×* time late—am. *×* time early	–0.000	–0.000	–0.000	–0.001
Am. late^2^—am. early^2^	(0.001) –0.001**	(0.001) –0.001	(0.001) –0.001	(0.001) –0.000
Time late^2^—time early^2^	(0.000) 0.002***	(0.000) 0.002***	(0.000) 0.002***	(0.000) 0.002***
	(0.000)	(0.000)	(0.000)	(0.000)
IQ	0.017**	0.017**	0.017**	0.017**
	(0.008)	(0.008)	(0.008)	(0.008)
First Lag		0.035***	0.106***	
		(0.008)	(0.016)	
Second Lag			–0.081***	
			(0.016)	
Late first, short lag				0.132**
				(0.064)
Late second, short lag				0.012
				(0.068)
Late second, long lag				0.396***
				(0.068)
Log Lik.	-2538.05	-2527.37	-2514.33	-2512.05
N	5111	5111	5111	5111

Models (2) to (4) consider the effect of the time lag (the time separation between the first and second option, and that of the separation between second option and joint presentation). In models (2) and (3) of the table, the variable *First Lag* is equal to *t*_1_×(*Ls*−*Lf*), where the function *Ls* (or *Lf*) is equal to 1 if the Late payment option is presented second (or first). The variable *Second Lag* is defined similarly, with *t*_2_ replacing *t*_1_. In model (4), the variable *Late first*, *short lag* is the indicator of the observations where the late option is presented first and the lag is short; the other variables are defined similarly; the variable *Late first*, *long lag* is taken as reference. In all four models we control for the second order terms (quadratic on the amount and the time delay, and the product of amount and time delay).

The marginal effect of First Lag indicates that an increase of the lag between the two presentations by one second changes the probability of choosing the option presented second (at equal value) by approximately 0.9 per cent. Model (4) indicates that the effect of the lag is non monotonic, flat for short lags, and substantial for longer lags. This finding indicates that the effect of the lag is due to the leak; it is not consistent with a model in which the only feature that matters is the order of presentation (that is, whether an option is presented first or second), and the length of the intervals is irrelevant.

Higher Intelligence induces, everything else being equal, a higher probability of choosing the late option: the marginal effect (at fixed means of the other variables) is 0.004, so an increase of the *IQ* score by one standard deviation (the standard deviation in our sample 15.7 points in the sample, mean *IQ* score = 113.7) is associated with an increase of that probability by 6.3 per cent. This finding is consistent with a large literature in psychology: see for example the meta-analysis of [16], who found that, across studies, higher intelligence was associated with lower Delay Discounting, defined as the tendency to prefer smaller, sooner rewards to larger, later ones. This confirms results in [15] for the same data set. The latter paper also shows that the response in caudate nucleus, part of a core evaluation network, to subjective value correlates with *IQ*, a result suggesting that the difference in behavior according to Intelligence is not entirely reducible to differences in information processing in choice.

Changes in probability of choice of one option associated with amount and time delay are as expected. Computing the marginal effects, we find that an increase by one dollar of the payment of an option increases the probability of choice of that option by approximately 1.9 per cent. The increase of the delay in the payment by one day reduces that probability by approximately 2.2 per cent. Note that the coefficients of the length of the two lags, First Lag and Second Lag in Table 1, model (3), are of opposite sign. This prediction follows if we impose a constraint on the model which is justified by a long tradition of the analysis of the reward evaluations.

### Response time

Important information on the choice process may be derived from the response time (*RT*), defined here as the length of the time interval between the time of joint presentation of the two options and the moment of choice by the participant. The distribution of *RT* is reported in Fig 1 it is approximately log-normal.

Considering the data of all participants, the mean of the *RT* is 830.3 *ms*, (*SD* = 636.3 *ms*, *max* = 5.7 *s*). There is a modest learning effect in the first run (5 *ms* reduction per trial) and a small fatigue effect in the second run (2 *ms* increase per trial).

The first important information we derive from the data of *RT* is that the decision is taken well before the threshold of 6*s*: all participants, in all trials, stay below that threshold; 98.7 per cent of the choices by all participants are taken within 3*s*, 94 per cent before 2*s* have elapsed.

To test the hypothesis that in the interval in which choice is allowed participants simply implement a choice that had been taken already, we test whether variables that may affect the reward evaluation component of the process of choice also affect *RT*: these are the absolute value of the difference between the subjective values of the two options (a smaller difference makes the decision harder), the intelligence score of the participant and the length of the interval between the display of the first and second option (a longer time interval should make the decision harder). If the choice at this stage is only the mechanical implementation of a choice already made, these variables should not be significant. The panel data regression of the standardized log of response time is reported in Table 2.

**Table 2:** Log of response time Independent variables: Absolute value of the subjective value difference and inter-offer interval. The subjective value is computed using the hyperbolic model. OLS. Standard error in parenthesis. All variables **except** the length of the inter-offer interval are standardized to mean 0 and SD 1.

	(1) b/se	(2) b/se	(3) b/se	(4) b/se
Absolute value of value difference	–0.070**	–0.069**	–0.076**	–0.076**
Length of inter-offer interval IQ	(0.013)	(0.013) –0.101	(0.013) 0.078** (0.011)	(0.013) 0.077** (0.011) –0.100
		(0.060)		(0.060)
Constant	0.002	0.002	–0.225**	–0.225**
	(0.060)	(0.060)	(0.069)	(0.069)
N	5165	5165	5165	5165

The variables have the expected sign, and they are either significant or marginally significant, and indicate sizable effects. In conclusion, since the mean response time is less than one second, it is likely that participants make a provisional choice before they see the two options displayed together. But since the response time depends in a significant and sizable way on three variables susceptible to reflect additional decision-making, such as the reappraisal of the options and change of mind, it is also likely that some final step in the choice is also taken after they see the two options displayed together.

Although they have been informed that the two options are going to be displayed again jointly, the influence of the delay between the two options on choices is direct proof that a provisional choice had been made after the presentation of the second option, and that the choice is not made from scratch after the joint presentation of the two options.

### Choice and performance in working memory task

Our second hypothesis is that the reduction of the probability of choosing the option presented first is larger in individuals with weaker working memory. In our model (see in particular the section on storage of information), the performance in the working memory task is inversely correlated with the leak parameter *ρ* (or, in more precise terms, the leak parameter *ρ*_*M*_). Thus, the model predicts that a higher value of the leak parameter is associated with a worse performance in the working memory task and a worse performance in the choice (stronger recency effect).

We test this hypothesis by comparing a measure of the effect of the presentation lag and a measure of performance in a working memory task. This task was completely independent of the choice. Performance on the task is summarized by combining the hit rate and the false alarm rate into a single score. A widely accepted way to combine the two results is a score, used here, known in psychometric literature as *d-prime* index: see [17].

**Fig 2:**
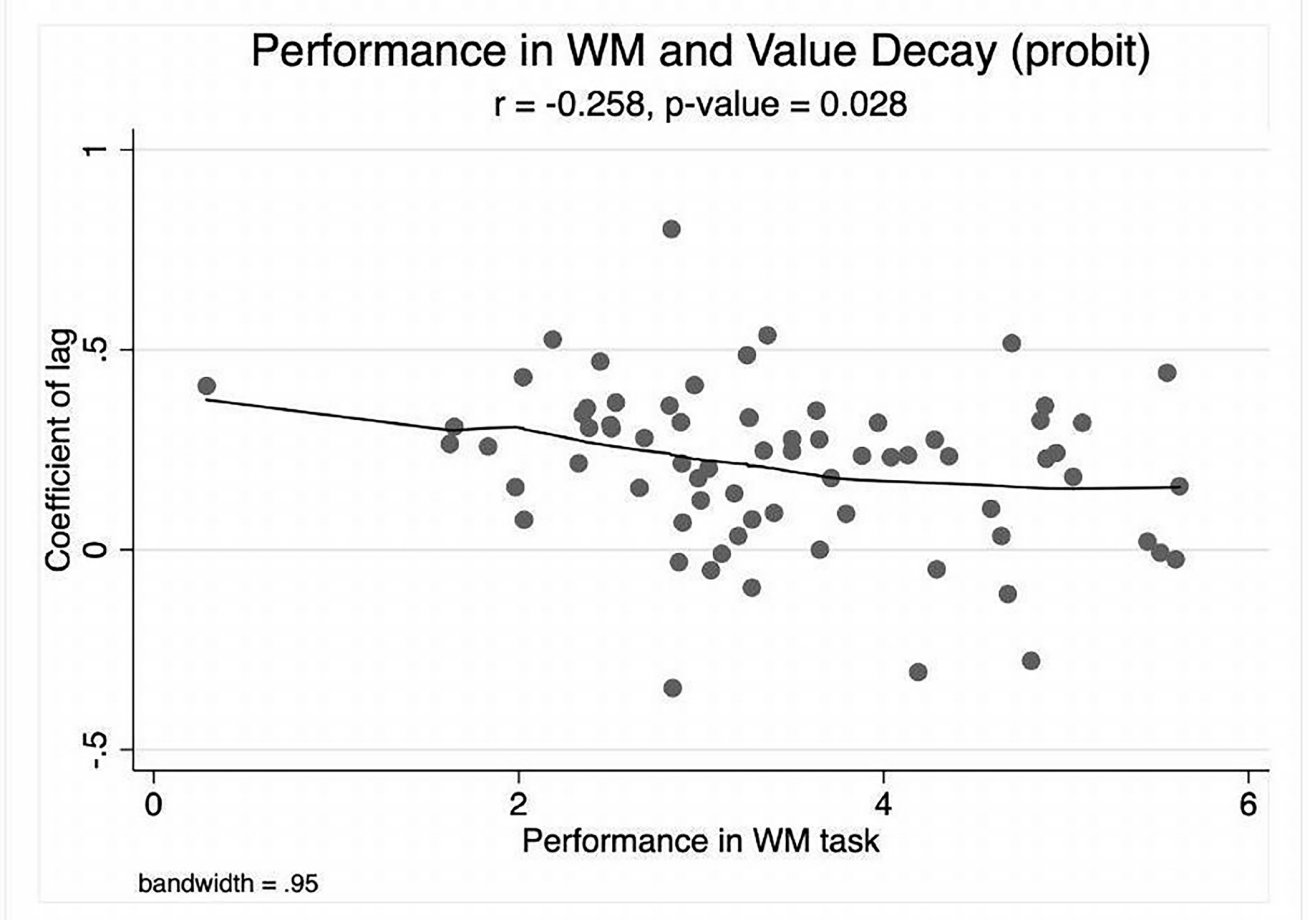
Working memory and memory of value. Horizontal axis: *d*-prime statistic. Vertical axis: estimated participant-by-participant coefficient in the probit regression of the lag on the probability of choice. Lowess and scatter-plot.

Our analysis offers support for the prediction: Fig 2 shows a negative correlation between the effect size of the decay in the stored value (measured by the estimated coefficient of the lag on the probability of choice) and the *d*-prime index of working memory performance.

To take into account the possible role of outliers we run a robust regression: the estimated coefficient of the dprime score has value -0.046, with robust *SE* = 0.018, *t* = −2.54, *p*-value = 0.013. The values for the OLS regression are as follows: estimated coefficient of the dprime score has value -0.046, with *SE* = 0.0208, *t* = −2.24, *p*-value = 0.028.

### Brain imaging results

If we want to test our sequential choice model the behavioral evidence we have examined so far only provides suggestive and indirect evidence, even if we consider data richer than choice data, taking into account for example response time and differences in intelligence scores and working memory performance. In particular we have seen only indirect evidence (the correlation of choice with the performance in the working memory task) suggesting that working memory is involved. The analysis of the evidence of the *fMRI* data can provide additional evidence to support the hypothesis.

### Predictions of the model for imaging data

The implications we describe follow naturally from the model presented in earlier sections; brain activity should correspond to the accumulation of evidence which follows the signal process that we have modeled. The hypothesis we formulate on the regions in which the activity should be observed follows from a body of knowledge that has been established, linking execution of reward evaluation tasks and patterns of brain activation, that we are going to recall briefly. The predicted sign of the effect follows the implications of the model.

1. (*Value Coding*.) The evaluation of first and second option should correspond to brain activation in reward areas, in particular striatum and *vmPFC*. Attention paid in the final interval, which according to the model should be concentrated on the option presented first, should also correspond to an activation in the *vmPFC*. Correlations between subjective values and BOLD activity in the orbito-frontal cortex are supported by meta-analysis over hundreds of fMRI studies on value-based choices ([18]);
2. (*Value Storage*.) The storage of value hypothesized should correspond to an activity in a network including parietal (Superior Parietal Lobule *PL*) (as well as inferior frontal gyrus (*IFG*) regions, proportionally to the value of the option considered as encoded in striatum and *vmPFC*. This prediction is consistent with the well known fact that *SPL* is involved in classical working memory task, such as the *n*-back used in our study to benchmark the general working memory performances of our participants ([19,20]). Finally, other brain regions classically involved with working memory ([21]) were also activated at the moment of presentation of the second option, when the subjective value of the first option is retrieved from *SPL* to *vmPFC*. In all these cases, the storage in memory is the product of an active process of maintenance of the information.
3. (Leaky Storage.) The decrease of the value stored should correspond to a pattern of decreasing activity in the working memory network (*SPL* and *IFG*).

### Whole-brain analysis

Image processing and statistical analysis were performed using *FEAT* (*fMRI* Expert Analysis Tool) Version 5.98, part of FSL (FMRIB’s Software Library). Registration to high-resolution structural and standard space images was carried out using FLIRT ([22]). A double-gamma *HRF* was used for convolution; temporal derivatives were added and temporal filtering was applied. A hierarchical general linear model was applied to the data: At the within-participant level, *Z* (Gaussianised T/F) statistic images were thresholded at *p* = 0.05 (uncorrected). For each participant, we included nine regressors in a *GLM*. The detailed description of the predictors in the GLM model is in section *S*−4.1.

Higher-level analysis, across participants, was carried out using the program FLAME ([23,24,25]). At this second level, *Z* (Gaussianised /*F*) statistic images were thresholded using clusters determined by *Z*>3.2, (*p*<0.001) and a (corrected) cluster significance threshold of *p* = 0.05.

### fMRI results

We now consider the technical details behind our claim that the pattern of brain activation provides support for our model of sequential choice. We rely on the analysis of a General Linear Model (*GLM*), a multivariate regression commonly adopted to model how independent variables (first among them the task that the participant is performing in that moment) affect the dependent variable. In imaging data analysis the dependent variable is the Blood-Oxygen-Level Dependent (*BOLD*). As brain activity increases, blood releases oxygen to neurons (through a process called hemodynamic response) at a rate greater than for inactive neurons. This flow of oxygen causes a change of the relative levels of oxygenated and deoxygenated blood. This level can be detected on the basis of their differential magnetic susceptibility, and is the BOLD signal. In our case, the *GLM* was designed to test our model of choice with sequential presentation of options; so it is focused on the evaluation of the first option, the activity during the first inter-stimulus interval and the evaluation of the second option.

We now examine the predictions on value coding, value storage and leaky storage.

### Value coding

As expected, we found that subjective value of the second option correlated positively with *BOLD* activity in the ventro-medial pre-frontal cortex at the onset of the second option (OMPFC, [26]), at 5% Family-Wise Error *FWE* corrected clusterwise. The *FWE* indicates the probability that a given study will produce any false positives when performing a multiple hypothesis test. See S5 Fig in S6 File section S-6 of the Appendix), the putative time of the provisional choice. The subjective value of the second option was also encoded in other brain regions including the striatum, the dorso-lateral prefrontal cortex, the lateral fronto-polar cortex, as well as posterior parietal cortex (see S2 Table in S2 File in section S-6.1 of the Supporting Information). We found weak (at conventional *p*-values) positive correlations between the subjective value of the first option and *BOLD* activity in the *vmPFC* at either the onset of the first or the second option, even after relaxing the threshold of our analysis to 0.005 voxelwise uncorrected. This may appear surprising in view of the large evidence we have reported earlier; but it is in line with the predictions we made on the attention effort, that increases with an exponential trend at rate *ρ*_*R*_.

We also note that an alternative *GLM* was estimated on a larger data set, including the data used for this paper. In this *GLM*, only the parametric regressor (i.e, the second regressor in the current model) was considered; in this case we found evidence of a linear correlation between the subjective value of the first option and the neural activation in striatum and dorsal anterior cingulate, among other areas ([15]). These results suggest that the subjective value of the first option is coded in brain areas that include reward-related areas, the striatum in the case of the entire data set.

In conclusion, the activity in the *vmPFC* we find at the moment of presentation of the first option appears weak or absent. This opens several interesting questions, some of which we have tried to address here, for example the need to test the hypothesis that modulation by attention of encoding of value may be important, particularly in setups like ours where options are presented sequentially.

### Value storage

The onset of the first option is associated with a significant activation in Right Precuneus and Right Middle Temporal Gyrus. As predicted by the model of choice, we observe then a decline of the activity in the Superior Parietal Lobule in the time interval after the presentation of the first option; this is illustrated in S4 Fig in S4 File. The decline is proportional to the length of time elapsed from the moment in which the first option is removed from the screen (top panel in S4 Fig S4 File). It is important to note that the decline is also larger, the larger the subjective value of the first option (bottom panel). This is consistent with a response in the working memory network at the moment of the presentation of the first option that is proportional to the value, and a subsequent decline from a higher value occurring at a faster speed.

### Leaky storage

Our behavioral findings suggest that there is temporal decay in the memory system maintaining the subjective value of the first option until the onset of the second; and this temporal decay correlates with participant performances in a classical working memory task. In view of these results we expected *BOLD* activity in this brain region to reflect this decay in the memory of subjective values. Consistent with our expectation, we found that BOLD activity in *SPL* decayed during the delay between the first and the second option (see S4 Fig in S4 File in section S-6 of the Appendix, top panel).

Crucially, we also found a correlation between *BOLD* activity in the *SPL* and subjective value of the option presented first. As expected from our behavioral findings, this correlation weakened over time (as shown by the interaction *time* × *subjective value of option 1*, see S4 Fig in S6 File section S-6 of the Appendix, bottom panel). Taken together these findings show that *SPL* maintains a working memory trace of the subjective of option 1 in the delay between the two options.

## Discussion

We consider an important contribution of the paper not specifically the identification of the behavioral recency effect, but rather its explanation in our experiment on the basis of a precise model, involving a specific form of memory (working memory), as the analysis of behavioral data and the correlation of performance in choice with performance in the working memory task confirm. The behavioral recency effect may very well depend on specific details of the experiment; for instance, changing the incentives to provision of attention effort might weaken or even reverse the effect. What would be common to the interpretation of these different experiments are the broad lines of the model, that puts testable constraints on the role of working memory in choice.

The paper introduced some innovations. First it provides a theoretical model of the way in which attention and working memory affect economic choices. The theoretical models of memory that are available in economic theory (most notably those emphasizing size constraints of memory) are conceptually inadequate for this purpose for the reasons we have seen in the Introduction. The theoretical model is not based on speculative normative criteria, but is biologically constrained by what we know of the psychometric properties and neural implementation of working memory. The model is experimentally tested, and validated with brain activity data.

The paper has also some limitations, and so there are natural extensions of the research we reported. Some are of secondary interest, and more a robustness check. For example, a new experiment where the two options are not presented together in the final stage could test the robustness of the recency effect in an environment which differs from ours only in this feature. As we argued earlier, the choice of presenting the two options together in the last step of the trail is very conservative. One may argue that if the options were not presented together in the last step, then it is obvious that the first option is at a disadvantage, just because it was shown earlier. The joint presentation allows us to conclude that the recency effect persists even if the two options have been observed by the participant, jointly and recently.

Interesting checks should involve more substantial modifications. Our model should explain different behavioral patterns in different environments, including possibly primacy effects, just as this explanation is possible in experiments on non-economic choices. A second more theoretical direction is an extension of existing biologically realistic models of choice in environments with simultaneous presentation of the two options (such as [9,28,29]). In particular, as we mentioned in the introduction, it would be interesting to extend the models in these references to include the interaction between memory and attention, as is examined in this paper, but within a setup closer to the biological constraints.

A third more general direction is a better understanding of the nature of choice with sequential presentation of options in animal subjects, not necessarily human. Our result, that the decision is in large part taken before the two options are again presented jointly, suggests that the choice process follows criteria which are different from optimal unconstrained information processing. With no constraint, optimal use of available information seems to be waiting for the last display, when all the details of the two options are again presented and are available for easy comparison. Instead, some constraints or different incentives acting perhaps through evolutionary selection pressures may have shaped a different, habitual information processing in the direction of preferably choosing sooner rather than later. For example, one may consider the decision process as in optimal foraging theory ([30]); in particular the process may be affected by the possible presence of competitors aiming at the same items ([31]). In this case, the gain from waiting for additional information should be weighted against the potential loss of the item to competitors, and this would justify the preferential use of the value stored in memory over future information to be collected in the next stage.

## Conclusion

We have provided a model of the role of attention and memory in economic choices where options are not necessarily presented simultaneously. In the model, choice is eventually the result of the comparison of the accumulated evidence in favor of the two options at a termination time which is set ex-ante. Attention modulates the rate of accumulation of the evidence; the evidence gathered in earlier stages decays with time. In view of this decay, attention devoted to an option increases over time at an exponential rate, unless constrained to a maximum value.

Our experimental evidence suggests that the option presented first is less likely to be chosen, everything else being equal. Also the size of the decay manifested in choice behavior correlates significantly (and negatively) with individual performance in a working memory task.

Behavioral and brain-imaging results provide evidence consistent with the following biologically realistic implementation of the attention and memory model. When the first option is presented, its value should be coded in reward-related areas. Since attention is lower at early stages, this coding may be weak. The value of the first option is then stored in working memory supported in a parietal region (*SPL*). In the time interval between the two presentations we observe a reduction of activity in parietal areas proportional to the value of the first option, which corresponds to a decrease of the value attributed to the first option in the direction of a common, non-informative baseline. At the moment of presentation of the second option its value is coded in *vmPFC*; the value of the first option is recalled in its reduced value, it is compared to the value of the first, and a choice is made.

The GLM analysis of brain imaging data we reported is consistent with this model, and with the view of working memory as emerging from the dynamic interaction of a network of brain regions including *vmPFC* and *SPL*. These findings are consistent with connectivity analysis showing that, during a delayed-response task, activity in sensory regions is correlated with activity in *PFC*, parietal cortex, striatum, and also the MTL ([27]). The decline of the stored value to a common non-informative baseline is supported by a model of the storage of value in working memory which is biologically realistic, is consistent with existing models of storage of information in visual perception, and predicts a decay to the non-informative baseline. Within the limitations mentioned in the discussion section, the theory provides an explanation of most of the important observed facts.

## Supporting information

S1 File Experimental design: Additional information. (DOCX)

S2 File Proofs. (DOCX)

S3 File Information gathering with Lévy processes. (DOCX)

S4 File Methods of FMRI data analysis. (DOCX)

S5 File Additional behavioral analysis results. (DOCX)

S6 File Additional FMRI results. (DOCX)

## References

[1] Cover T, Hellman M. The two-armed-bandit problem with time-invariant finite memory. IEEE Transactions on Information Theory. 1970 Mar;16(2):185–95.

[2] Wilson A. Bounded memory and biases in information processing. Econometrica. 2014 Nov;82(6):2257–94.

[3] Glautier S. Recency and primacy in causal judgments: Effects of probe question and context switch on latent inhibition and extinction. Memory & cognition. 2008 Sep;36(6):1087–93. doi: 10.3758/MC.36.6.1087 18927027

[4] Kable JW, Glimcher PW. The neural correlates of subjective value during intertemporal choice. Nature neuroscience. 2007 Dec;10(12):1625–33. doi: 10.1038/nn2007 17982449 PMC2845395

[5] Ballard K, Knutson B. Dissociable neural representations of future reward magnitude and delay during temporal discounting. Neuroimage. 2009 Mar 1;45(1):143–50. doi: 10.1016/j.neuroimage.2008.11.004 19071223 PMC2685201

[6] Tremblay L, Schultz W. Relative reward preference in primate orbitofrontal cortex. Nature. 1999 Apr;398(6729):704–8. doi: 10.1038/19525 10227292

[7] Fiorillo CD, Tobler PN, Schultz W. Discrete coding of reward probability and uncertainty by dopamine neurons. Science. 2003 Mar 21;299(5614):1898–902. doi: 10.1126/science.1077349 12649484

[8] Padoa-Schioppa C, Assad JA. Neurons in the orbitofrontal cortex encode economic value. Nature. 2006 May;441(7090):223–6. doi: 10.1038/nature04676 16633341 PMC2630027

[9] Rustichini A, Padoa-Schioppa C. A neuro-computational model of economic decisions. Journal of neurophysiology. 2015 Sep;114(3):1382–98. doi: 10.1152/jn.00184.2015 26063776 PMC4556855

[10] Krajbich I, Armel C, Rangel A. Visual fixations and the computation and comparison of value in simple choice. Nature neuroscience. 2010 Oct;13(10):1292–8. doi: 10.1038/nn.2635 20835253

[11] Tavares G, Perona P, Rangel A. The attentional drift diffusion model of simple perceptual decision-making. Frontiers in neuroscience. 2017 Aug 24;11:468. doi: 10.3389/fnins.2017.00468 28894413 PMC5573732

[12] Andersen S, Harrison GW, Lau MI, Rutström EE. Eliciting risk and time preferences. Econometrica. 2008 May;76(3):583–618.

[13] Webb R. The (neural) dynamics of stochastic choice. Management Science. 2019 Jan; 65(1):230–55.

[14] Smith SM, Krajbich I, Webb R. Estimating the dynamic role of attention via random utility. Journal of the Economic Science Association. 2019 Aug;5(1):97–111.

[15] Civai C, Hawes DR, DeYoung CG, Rustichini A. Intelligence and extraversion in the neural evaluation of delayed rewards. Journal of Research in Personality. 2016 Apr 1;61:99–108.

[16] Shamosh NA, Gray JR. Delay discounting and intelligence: A meta-analysis. Intelligence. 2008 Jul 1;36(4):289–305.

[17] Snodgrass JG, Corwin J. Pragmatics of measuring recognition memory: applications to dementia and amnesia. Journal of experimental psychology: General. 1988 Mar;117(1):34. doi: 10.1037//0096-3445.117.1.34 2966230

[18] Clithero JA, Rangel A. Informatic parcellation of the network involved in the computation of subjective value. Social cognitive and affective neuroscience. 2014 Sep 1;9(9):1289–302. doi: 10.1093/scan/nst106 23887811 PMC4158359

[19] Duncan J. The multiple-demand (MD) system of the primate brain: mental programs for intelligent behaviour. Trends in cognitive sciences. 2010 Apr 1; 14(4): 172–9. doi: 10.1016/j.tics.2010.01.004 20171926

[20] Postle BR. Working memory as an emergent property of the mind and brain. Neuroscience. 2006 Apr 28;139(1):23–38. doi: 10.1016/j.neuroscience.2005.06.005 16324795 PMC1428794

[21] Eriksson J, Vogel EK, Lansner A, Bergström F, Nyberg L. Neurocognitive architecture of working memory. Neuron. 2015 Oct 7;88(1):33–46. doi: 10.1016/j.neuron.2015.09.020 26447571 PMC4605545

[22] Jenkinson M, Bannister P, Brady M, Smith S. Improved optimization for the robust and accurate linear registration and motion correction of brain images. Neuroimage. 2002 Oct 1;17(2):825–41. doi: 10.1016/s1053-8119(02)91132-8 12377157

[23] Beckmann CF, Jenkinson M, Smith SM. General multilevel linear modeling for group analysis in FMRI. Neuroimage. 2003 Oct 1;20(2):1052–63. doi: 10.1016/S1053-8119(03)00435-X 14568475

[24] Woolrich MW, Behrens TE, Beckmann CF, Jenkinson M, Smith SM. Multilevel linear modelling for FMRI group analysis using Bayesian inference. Neuroimage. 2004 Apr 1;21(4):1732–47. doi: 10.1016/j.neuroimage.2003.12.023 15050594

[25] Woolrich M. Robust group analysis using outlier inference. Neuroimage. 2008 Jun 1;41(2):286–301. doi: 10.1016/j.neuroimage.2008.02.042 18407525

[26] Öngür D, Price JL. The organization of networks within the orbital and medial prefrontal cortex of rats, monkeys and humans. Cerebral cortex. 2000 Mar 1; 10(3):206–19. doi: 10.1093/cercor/10.3.206 10731217

[27] Gazzaley A, Rissman J, D’esposito M. Functional connectivity during working memory maintenance. Cognitive, Affective, & Behavioral Neuroscience. 2004 Dec;4(4):580–99. 10.3758/cabn.4.4.580 15849899

[28] Wong KF, Wang XJ. A recurrent network mechanism of time integration in perceptual decisions. Journal of Neuroscience. 2006 Jan 25;26(4):1314–28. doi: 10.1523/JNEUROSCI.3733-05.2006 16436619 PMC6674568

[29] Rustichini A, Conen KE, Cai X, Padoa-Schioppa C. Optimal coding and neuronal adaptation in economic decisions. Nature communications. 2017 Oct 31;8(1):1–4. 10.1038/s41467-017-01373-y PMC5662730 29084949

[30] Charnov EL. Optimal foraging, the marginal value theorem. Theoretical population biology. 1976 Apr 1;9(2):129–36. doi: 10.1016/0040-5809(76)90040-x 1273796

[31] Milinski M. Optimal foraging: the influence of intraspecific competition on diet selection. Behavioral Ecology and Sociobiology. 1982 Oct;11(2):109–15.

