# Supplementary material for: Working memory and attention in choice
Source: PLoS One. 2023 Oct 11;18(10):e0284127. doi: 10.1371/journal.pone.0284127 (PMC10566694; doi:10.1371/journal.pone.0284127)
Supplement: S1 File — (DOCX) [file pone.0284127.s001.docx]

**S-1 Experimental Design: Additional Information**

***S-1.1 Participants***

Our analysis relies on data from a sample of 96 participants who had a random balanced allocation of early and late payment options as presented first or second. This sample is part of a larger study with $N=248$ (number of females $=125$, age: mean $=26.52$, sd $=4.68)$. The set of participants we do not consider here saw the early payment option always presented first, in a design aimed at testing a different hypothesis ( [15]).

To increase the diversity of our participants pool with respect to socio-economic background, we recruited participants through a regional classified advertisements web-site (Craigslist). We paid participants $\$25$ dollars per hour in the behavioral task, and $\$50$ dollars per hour in the scanning section of the study, plus any amount gained in the task.

The study did not include minors. The participants in the study provided written consent to participate. The ethical committee at the University of Minnesota approved of the study (protocol number *IRB 1002M78152*).

***S-1.2 Tasks in the Experiment***

The choice experiment was part of a wider study, which was aiming at identifying features of the personality of participants and economic behavior. In the $MRI$ session, participants performed three types of tasks:

1. $n$-back task, with $n$ equal to 1 and 3: participants were presented with a sequence of letters one-by-one; for each letter, they had to decide whether the current letter was the same as the one presented 1 (1-back) or 3 trials ago.
2. A choice of lottery task, where participants were asked to choose between two lotteries with different levels of risk.
3. A choice among time payments, described in the main text.

The session ended with a Diffusion-Weighted Imaging (DWI) measurement, lasting 20 minutes, while participants were watching a relaxing video. Overall, the MRI session lasted 2 hours, including a short training for each task before entering the scanner.

***S-1.3 Procedure and Timeline of the design***

Participants completed two experimental sessions. The first session of behavioral assessments lasted 5 hours. In this first session participants were administered different personality questionnaires. Two weeks later, participants were recalled for a session of functional Magnetic Resonance Imaging ( $fMRI$ ), which lasted in total on average $2\frac{1}{2}$ hours. During the second session, participants lay supine in the $MR$ scanner for approximately 1 hour and $20min(5$ minutes required for the anatomical scans, and the rest divided among 6 different tasks). The experimental task was presented using E-prime 2.0 software (Psychology Software Tools, Inc.); a Sanyo projector mounted outside of the room projected through a waveguide to a screen placed in the bore of the scanner behind the participant's head.

***S-1.4 Personality Questionnaires and Cognitive Abilities***

In the first session (a few days before the scanning session), participants took part in a behavioral experiment session. Intelligence was estimated using four sub-tests of the Wechsler Adult Intelligence Scale, Fourth Edition (*WAIS-IV*).
