# Supplementary material for: Working memory and attention in choice
Source: PLoS One. 2023 Oct 11;18(10):e0284127. doi: 10.1371/journal.pone.0284127 (PMC10566694; doi:10.1371/journal.pone.0284127)
Supplement: S2 File — (DOCX) [file pone.0284127.s002.docx]

**S-2 Proofs**

**Proof of Proposition 7**

Existence of an optimal solution follows from standard arguments. By assumption 3 the cost functional is convex. Note $\sum_{o} G\left( o \right)\geq0$; we consider the non-trivial case in which there exists at least one state which has positive $\pi$ probability for which the utility of the two options is different. We prove that this implies $\sum_{o} G\left( o \right)>0$.

The leaked process for an attention policy pair $\left( a\left( \cdot,A \right),a\left( \cdot,B \right) \right)$, obtained combining equations (13) and (14), is for option $o$ :

(S-1)

$LX\left( T;\theta,o,a\left( \cdot,o \right) \right)=\int_{0}^{T} e^{-\rho\left( T-s \right)}\left( u\left( \theta,o \right)a\left( s,o \right)ds+\sigma dW\left( s \right) \right)$

and thus substituting into the definition (11), the expected utility in our case is:

(S-2)

$E_{a,\pi}U= \sum_{\theta,o} \pi\left( \theta\right)u\left( \theta,o \right)P\left( \{LX\left( T;\theta,o,a\left( \cdot,o \right) \right)>LX(T;\theta,o^{'},a\left( \cdot,o^{'} \right)\}) \right) .$

We rewrite $E_{a,\pi}U$ as

(S-3)

$\sum_{\theta} \pi\left( \theta\right)u\left( \theta,B \right)+ \sum_{\theta} \pi(\theta)(u(\theta,A)-u(\theta,B))Pr(\{LX(T;\theta,A,a(\cdot,A)>LX(T;\theta,B,a(\cdot,B)\})$

The term $\sum_{\theta} \pi\left( \theta\right)u\left( \theta,B \right)$ is independent of the attention and the decision time, so we ignore it in looking for an optimal policy and consider only the second addend in (S-3).

We write $V\left( a,T \right)$ the functional to be maximized on feasible $\left( a,T \right)$ pairs of attention effort paths and stopping time $T$. Substituting (S-1) into the second addend of (S-3) and using equations (14) and (15), and differentiating we get that the derivatives with respect to the terms $a\left( s,o \right)$ are, for each option $o$ :

(S-4)

$\frac{\partial V}{\partial a\left( s,o \right)}=e^{\rho s}Q\left( T,o \right)-c^{'}\left( \sum_{b} a\left( s,b \right) \right),s\in\left[ 0,t_{F} \right]$

where we have defined the difference in total drift between the two options, normalized by the variance of the noise, as:

(S-5)

$D\left( \theta\right)\equiv\frac{1}{\sigma\left( \rho,T \right)}\left( u\left( \theta,A \right)\int_{0}^{T} e^{-\rho\left( T-r \right)}a\left( r,A \right)dr-u\left( \theta,B \right)\int_{0}^{T} e^{-\rho\left( T-r \right)}a\left( r,B \right)dr \right);$

and, for each option $o$ :

(S-6)

$Q\left( T,o \right)\equiv\sum_{\theta} \pi\left( \theta\right)\left( u\left( \theta,o \right)-u\left( \theta,o^{'} \right) \right)\phi\left( D\left( \theta\right) \right)\frac{e^{-\rho T}}{\sigma\left( \rho,T \right)}u\left( \theta,o \right)$

where $\phi$ is the density of a standard normal. Note that both $D\left( \theta\right)$ and $Q\left( T,o \right)$ for both options $o$ are independent of the time $s$.

From the definition $(S-6)$ we derive:

(S-7)

$\sum_{o} Q(T,o)=\sum_{\theta} \pi(\theta)(u(\theta,A)-u(\theta,B))^{2}\phi(D(\theta))\frac{e^{-\rho T}}{\sigma\left( \rho,T \right)}$

If for all $\theta,u\left( \theta,A \right)=u\left( \theta,B \right)$ then paying no attention and choosing with arbitrary probability is optimal. If not, then from our assumption that there are no zero-probability states, and the fact that the normal density is everywhere strictly positive, we conclude that $\sum_{o} Q\left( T,o \right)>0$, and therefore at least one of the two values $Q\left( T,o \right)$, for $o\in A,B$ is strictly positive. Hence our claim follows.

We consider paths that satisfy the first order conditions applied to the Lagrangean with multipliers $\lambda_{o},o\in\{A,B\}$. Existence of an optimal solution follows from standard arguments. We consider paths that satisfy the first order conditions applied to the Lagrangean with multipliers $\lambda_{o},o\in\{A,B\}.c^{'}$ is the derivative of the cost function.

The following first order conditions hold:

(S-8)

$c^{'}\left( \sum_{o} a\left( s,o \right) \right)-\lambda_{o}e^{-\rho\left( T-s \right)}\geq0,s\in\left[ 0,T \right],o\in A,B$

(S-9)

$\left( c^{'}\left( \sum_{o} a\left( s,o \right) \right)-\lambda_{o}e^{-\rho\left( T-s \right)} \right)a\left( s,o \right)=0,s\in\left[ 0,T \right],a\in A,B,$

We first prove that if $G\left( o \right)>0$ for both $o$, then:

(S-10)

$\lambda_{A}=\lambda_{B}\equiv\lambda$

Suppose instead that the largest of the two, $\lambda_{o}$ is strictly larger than the other, $\lambda_{o^{'}}$. The inequalities (S-9) and our contradiction hypothesis imply:

(S-11)

$c^{'}\left( \sum_{o} e\left( s,o \right) \right)\geq\lambda_{o}e^{-\rho\left( T-s \right)}>\lambda_{o^{'}}e^{-\rho\left( T-s \right)}$

and now the strict inequality in (S-11) and the condition $\left( S-9 \right)$ applied to $o^{'}$ imply $a\left( s,o^{'} \right)=0$ for all $s\in\left[ 0,T \right]$, contradicting the assumption that $G\left( o \right)>0$ for both $o$.

Let ${\sum_{o} a\left( \cdot,o \right)|}_{\left[ 0,s \right]}$ denote the restriction of the function to the interval $\left[ 0,s \right]$. Define

(S-12)

$S‾\equiv\left\{ s\in\left[ 0,T \right]: ess sup\left( {\sum_{a} a\left( \cdot,o \right)|}_{\left[ 0,s \right]} \right)>0 \right\}$

(S-13)

$\underline{S}\equiv\left\{ s\in\left[ 0,T \right]: ess sup\left( {\sum_{o} a\left( \cdot,o \right)|}_{\left[ 0,s \right]} \right)=0 \right\}$

and let:

(S-14)

$s‾\equiv inf\left( S‾ \right),\underline{s}\equiv sup\left( \underline{S} \right)$

If $s\in S‾,t\in\underline{S}$, then $s\geq t$, because $t>s$ implies a contradiction. Thus, $s‾\geq\underline{s}$. But the hypothesis $s‾>\underline{s}$ would imply the existence of $t\in\left( \underline{s},s‾ \right)$, for which ess ${sup\left( \sum_{o} a\left( \cdot,o \right) \right)|}_{\left[ 0,t \right]}$ cannot be either $=0$ or $>0$; thus we conclude:

(S-15)

$\underline{s}=s‾$

Now note that

(S-16)

$\underline{s}<T$

because equality would imply that

$\int_{0}^{T} \sum_{o} a\left( s,o \right)ds=0$

contradicting the assumption that $\sum_{o} G\left( o \right)>0$. Pick now any $sˆ\in\left( \underline{s},T \right)$ such that

(S-17)

$\sum_{o} a\left( sˆ,o \right)>0$

We note immediately that by $\left( S-16 \right)$ such element exists, and by $(S-15)$ it can be picked arbitrarily close to $\underline{s}$. Using $\left( S-9 \right),\left( S-10 \right)$ and $\left( S-17 \right)$ we derive:

(S-18)

$c^{'}\left( \sum_{o} a\left( sˆ,o \right) \right)=\lambda e^{-\rho\left( T-sˆ \right)}.$

Consider now any $t\geq sˆ$; we have:

$c^{'}\left( \sum_{o} a\left( t,o \right) \right) \geq\lambda e^{-\rho\left( T-t \right)} \geq\lambda e^{-\rho\left( T-sˆ \right)} =c^{'}\left( \sum_{o} a\left( sˆ,o \right) \right).$

because of (S-9), $t\geq sˆ$ and (S-18) respectively. By convexity of the function $c$, we conclude:

(S-19)

$\sum_{o} a\left( t,o \right)\geq\sum_{o} a\left( sˆ,o \right).$

In particular, using (S-17), (S-19), and our earlier comment that $sˆ$ can be chosen arbitrarily close to $\underline{s}$, we derive:

(S-20)

$\forall t\in\left( \underline{s},T \right),\sum_{o} a\left( t,o \right)>0.$

and therefore using $\left( S-9 \right)$ we conclude the last statement:

$\forall s\in\left( \underline{s},T \right):c^{'}\left( \sum_{a} a\left( s,o \right) \right)=\lambda e^{-\rho\left( T-s \right)}$

**Proof of Proposition 8**

The optimal efforts are characterized by the following lemma 14. The statements (1), (3) and (4) below are symmetric for the case of $I_{A}$ and $I_{B}$. For example, the statement (1) below has the correspondent for option $B$ stated as: If $s,t\in I_{B}$, then $a\left( t,B \right)\geq a\left( s,B \right)$; and if $a\left( s,B \right)>0$, then

$c^{'}\left( a\left( t,B \right) \right)=c^{'}(a\left( s,AB \right)e^{\rho\left( t-s \right)}.$

so we present them only for $I_{A}$.

**Lemma 14** *Take any pair of times* $s,t\in\left[ 0,t_{F} \right]$*, with* $t>s$*. Then:*

1. *If* $s,t\in I_{A}$*, then* $a\left( t,A \right)\geq a\left( s,A \right)$*; and if* $a\left( s,A \right)>0$*, then*

(S-21)

$c^{'}\left( a\left( t,A \right) \right)=c^{'}\left( a\left( s,A \right) \right)e^{\rho\left( t-s \right)}.$

1. *If* $s,t\in I_{A,B}$*, then* $\sum_{o} a\left( t,o \right)\geq\sum_{o} a\left( s,o \right)$*; and if* $\sum_{o} a\left( s,o \right)>0$*, then*

(S-22)

$c^{'}\left( \sum_{o} a\left( t,o \right) \right)=c^{'}\left( \sum_{o} a\left( s,o \right) \right)e^{\rho\left( t-s \right)}.$

1. *If* $s\in I_{A},t\in I_{A,B}$*, then* $\sum_{o} a\left( t,o \right)\geq a\left( s,A \right)$*; if* $a\left( s,A \right)>0$*, then*

(S-23)

$c^{'}\left( \sum_{o} a\left( t,o \right) \right)\geq c^{'}\left( a\left( s,A \right) \right)e^{\rho\left( t-s \right)}.$

$If c^{'}\left( \sum_{o} a\left( t,o \right) \right)>c^{'}\left( a\left( s,A \right) \right)e^{\rho\left( t-s \right)}, then a\left( t,A \right)=0;$

1. *If* $s\in I_{A,B},t\in I_{A}$*, then* $a\left( t,A \right)\geq\sum_{o} a\left( s,o \right)$*; and if* $\sum_{o} a\left( s,o \right)>0$*, then*

(S-24)

$c^{'}\left( a\left( t,A \right) \right)\geq c^{'}\left( \sum_{o} a\left( s,o \right) \right)e^{\rho\left( t-s \right)}.$

The proof of lemma 14 is as follows. Define the Lagrangean for the problem (18) and (22), and call $\lambda_{A}$ and $\lambda_{B}$ the multipliers for the two constraints. Consider first (1), and assume $s,t\in I_{A}$. If $a\left( s,A \right)=0$ then the conclusion is obvious. If $a\left( s,A \right)>0$, then

$c^{'}\left( a\left( t,A \right) \right) \geq\lambda_{A}e^{-\rho\left( T-t \right)} =\lambda_{A}e^{-\rho\left( T-s \right)}e^{\rho\left( t-s \right)} =c^{'}\left( a\left( s,A \right) \right)e^{\rho\left( t-s \right)} >c^{'}\left( a\left( s,A \right) \right),$

where the first inequality follows from the first order condition, the second in clear, the third from the complementary slackness and the condition $a\left( s,A \right)>0$, the fourth from $t>s$ and the condition $a\left( s,A \right)>0$ again. We conclude by convexity of $c$ that $a\left( t,A \right)\geq a\left( s,A \right)$ and therefore $a\left( t,A \right)>0$. Using the complementary slackness condition at $t$ we conclude that the first inequality is in fact an equality, and hence $c^{'}\left( a\left( t,A \right) \right)=c^{'}\left( a\left( s,A \right) \right)e^{\rho\left( t-s \right)}$ as claimed. This concludes the proof of (1).

We consider next the proof of (2). If $\sum_{o} a\left( s,o \right)=0$ then the conclusion is obvious. If $\sum_{o} a(s,o)>0($ say, $a(s,A)>0)$ then

$c^{'}\left( \sum_{o} a\left( t,o \right) \right) \geq max\left\{ \lambda_{A},\lambda_{B} \right\}e^{-\rho\left( T-t \right)} =max\left\{ \lambda_{A},\lambda_{B} \right\}e^{-\rho\left( T-s \right)}e^{\rho\left( t-s \right)} =c^{'}\left( \sum_{o} a\left( s,o \right) \right)e^{\rho\left( t-s \right)} \geq c^{'}\left( \sum_{o} a\left( s,o \right) \right).$

This implies $\sum_{o} a\left( t,o \right)>0$ as in the previous case, and hence the claimed equality follows by the complementary slackness condition.

For (3), if $a\left( s,A \right)=0$ then the conclusion is obvious. If $a\left( s,A \right)>0$

$c^{'}\left( \sum_{o} a\left( t,o \right) \right) \geq max\left\{ \lambda_{A},\lambda_{B} \right\}e^{-\rho\left( T-t \right)} =max\left\{ \lambda_{A},\lambda_{B} \right\}e^{-\rho\left( T-s \right)}e^{\rho\left( t-s \right)} \geq\lambda_{A}e^{-\rho\left( T-s \right)}e^{\rho\left( t-s \right)} =c^{'}\left( a\left( s,A \right) \right)e^{\rho\left( t-s \right)} >c^{'}\left( a\left( s,A \right) \right)$

by the usual arguments, hence $\sum_{o} a\left( t,o \right)\geq a\left( s,A \right)$ by convexity of $c$. By complementary slackness, we conclude:

$c^{'}\left( \sum_{o} a\left( t,o \right) \right)=max\left\{ \lambda_{A},\lambda_{B} \right\}e^{-\rho\left( T-t \right)}$

If $\lambda_{A}\geq\lambda_{B}$ then

$c^{'}\left( \sum_{o} a\left( t,o \right) \right)=c^{'}\left( a\left( s,A \right) \right)e^{-\rho\left( T-t \right)}$

If instead $\lambda_{B}>\lambda_{A}$, then the complementary slackness conditions imply $\sum_{o} a\left( t,o \right)=a\left( t,B \right)$. The proof of (4) is similar.

**Proof of Proposition 9**

The proof follows the lines of the first part of the proof of Proposition 7, with some obvious modifications. The feasibility constraint in the case we are considering requires that no attention can be devoted to an option which is not displayed requires $a\left( s,B \right)=0$ in $s\in[0,t_{1})$ and $a\left( s,A \right)=0$ in $s\in[t_{1},t_{2})$. Thus, for instance, the equation (S-4) in the current case holds only for $s\in I_{o}\cup I_{o,o^{'}}$ rather than $s\in\left[ 0,t_{F} \right]$.

The first order conditions are given by the usual inequalities and complementary slackness for derivatives as in equation (S-4). Using the power form of the cost we get (24) and (25) when effort is positive. In this case we set

(S-25)

$x_{1}=\left( \frac{Q\left( T,A \right)}{R} \right)^{\frac{1}{R-1}},y_{1}=\left( \frac{Q\left( T,B \right)}{R} \right)^{\frac{1}{R-1}}.$

The same argument shows the existence of $\left( x_{2},y_{2} \right)$.

**Proof of Lemma 10**

Since the two noise processes of the two options are independent, by a well known property of the Ornstein-Uhlenbeck process we conclude that the function is:

$\sigma^{2}\left( \rho,T \right)=\frac{\sigma^{2}}{\rho}\left( 1-e^{-2\rho T} \right)$

this is clearly increasing in $T$ when $\rho>0$. Its limit at zero leak rate is:

(S-26)

$\lim_{\rho\to0} \frac{\sigma^{2}}{\rho}\left( 1-e^{-2\rho T} \right)\equiv\sigma^{2}\left( 0,T \right)=2\sigma^{2}T$

as natural, because this is the variance of the sum of two independent Brownian motions with no decay.

**Proof of Proposition 11**

Let $\Phi$ denote the cumulative distribution of the standard normal, and $\phi$ the density. The value function $V$ on $\left[ 0,a‾ \right]\times\left[ t_{2},t_{F} \right]$ is defined in (31), and is equal to:

$V\left( a,T \right) =2\Phi\left( \frac{E\left( a,T \right)}{\sigma\left( 0,T \right)} \right)-c\left( a \right)T =2\Phi\left( \frac{aT^{1/2}}{\sqrt{2}\sigma} \right)-c\left( a \right)T.$

Denoting $V_{a}$ derivative with respect to the common value $a$ and with $V_{T}$ the derivative with respect to $T$, we get:

(S-27)

$V_{T}\left( a,T \right)=2\phi\left( \frac{aT^{1/2}}{\sqrt{2}\sigma} \right)\frac{1}{\sqrt{2}\sigma T^{1/2}}\frac{a}{2}-c\left( a \right) =\left( 2\phi\left( \frac{aT^{1/2}}{\sqrt{2}\sigma} \right)\frac{1}{\sqrt{2}\sigma T^{1/2}}-\frac{2c\left( a \right)}{a} \right)\frac{a}{2}$

and

(S-28)

$V_{a}\left( a,T \right)=\left( 2\phi\left( \frac{aT^{1/2}}{\sqrt{2}\sigma} \right)\frac{1}{\sqrt{2}\sigma T^{1/2}}-c^{'}\left( a \right) \right)T$

Now consider the function:

(S-29)

$\frac{2c\left( a \right)}{a}-c^{'}\left( a \right)=\left( 2-R \right)a^{R-1}.$

The sign of this function is constant independent of $a$, and for any $a>0$ it is the same as the sign of $2-R$. The conclusions follow.

**Proof of Corollary 12**

An interior solution (that is a pair $\left( aˆ,Tˆ \right)\in\left( 0,a‾ \right)\times\left( t_{2},t_{F} \right)$ with both derivatives equal to $0)$ is possible only if $c^{'}\left( aˆ \right)=c\left( aˆ \right)\frac{2}{aˆ}$, and this is the case with $aˆ>0$ if and only if $R=2$.

If $R\in\left( 1,2 \right)$, then in particular $c\left( aˆ \right)\frac{2}{aˆ}>c^{'}\left( aˆ \right)$ if $aˆ>0$, and so $V_{T}\left( aˆ,Tˆ \right)\geq0$ implies $V_{a}\left( aˆ,Tˆ \right)>0$, and therefore $aˆ=a‾$.

If $R\in\left( 2,+\infty\right)$, then in particular $c\left( aˆ \right)\frac{2}{aˆ}<c^{'}\left( aˆ \right)$ if $aˆ>0$, and so $V_{a}\left( aˆ,Tˆ \right)\geq0$ implies $V_{T}\left( aˆ,Tˆ \right)>0$, and therefore $Tˆ=t_{F}$.

**Proof of Proposition 13**

We begin with the case in which the two paths are interior, for instance when $\underline{a}=0$ and $a‾=+\infty$.

Under the assumptions of the proposition, and the characterization offered in proposition 9 the maximization of net utility is equivalent to the choice of the two values $x_{1}$ and $y_{1}$. Using the exponential form of the optimal policies in equations (24) and (25), the maximization is equivalent to:

(S-30)

$\max_{x_{1},y_{1}} \frac{1}{2}(Pr\left( x_{1}K_{1}+W_{\rho}^{A}\left( T \right)>W_{\rho}^{B}\left( T \right) \right) + Pr\left( y_{1}e^{\rho t_{1}}K_{1}+W_{\rho}^{B}\left( T \right)>W_{\rho}^{A}\left( T \right) \right) )-x_{1}^{R}K_{2}-y_{1}^{R}K_{2}.$

where we have defined:

(S-31)

$K_{1}\equiv e^{-\rho t_{2}}\int_{0}^{t_{1}} e^{\left( \rho_{R}+\rho\right)s}ds;K_{2}\equiv\int_{0}^{t_{1}} e^{\rho_{R}Rs}ds.$

We have used the fact that $T=t_{2}$ and $t_{1}=t_{2}-t_{1}$.

Recall that:

$\sigma^{2}\left( \rho,T \right)$

$Pr\left( x_{1}K_{1}+W_{\rho}^{A}\left( T \right)>W_{\rho}^{B}\left( T \right) \right) =\Phi\left( \frac{x_{1}K_{1}}{\sigma\left( \rho,T \right)} \right).$

The maximization over the two variables $x_{1}$ and $y_{1}$ in the problem (S-30) can be performed separately, so that the problem (S-30) is equivalent to the two problems:

(S-32)

$\max_{x_{1}} \left( \Phi\left( \frac{x_{1}K_{1}}{\sigma\left( \rho,T \right)} \right)-x_{1}^{R}K_{2} \right)$

and

(S-33)

$\max_{y_{1}} \left( \Phi\left( \frac{y_{1}K_{1}e^{\rho t_{1}}}{\sigma\left( \rho,T \right)} \right)-y_{1}^{R}K_{2} \right)$

Note that the two problems are similar: the only difference is that in the maximization over $y_{1}$ the value of $K_{1}$ is multiplied by a factor larger than 1 . Calculus shows that the inequality (32) reported here for convenience:, at the two optimal values ${xˆ}_{1}$ and ${yˆ}_{1}$ :

${yˆ}_{1}e^{\rho t_{1}}>{xˆ}_{1}$

follows, when $\rho>0$. The claim of the proposition (that the option presented second is more likely to be chosen, in spite of the symmetry) is equivalent to (32). The proof for the other cases is obvious.
