# Supplementary material for: Working memory and attention in choice
Source: PLoS One. 2023 Oct 11;18(10):e0284127. doi: 10.1371/journal.pone.0284127 (PMC10566694; doi:10.1371/journal.pone.0284127)
Supplement: S3 File — (DOCX) [file pone.0284127.s003.docx]

**S-3 Information Gathering with Lévy Processes**

The purpose of this section is to show that the essential points of the analysis that we presented in the main text can be extended to more general stochastic processes, Lévy processes. We first recall the basic definition:

**Definition 15** A *stochastic process on a probability space* $\left( \Omega,F,P \right)$ *with values on* $R^{d}$*,* $\left( X_{t}:t\geq0 \right)$ *is a Lévy process if and only if:*

1. *For any* $n\in N,n\geq1$ *and* $0\leq t_{0}<t_{1}<\cdots<t_{n}$*, the random variables* $X_{t_{0}},X_{t_{1}}-X_{t_{0}},\ldots,X_{t_{n}}-X_{t_{n-1}}$ *are independent*
2. $X_{0}=0,P$*-a.s.;*
3. *For every* $s,t$*, the distribution of* $X_{s+t}-X_{s}$ *does not depend on* $s$*;*
4. *The process is stochastically continuous;*
5. *The process has* $P$*-almost surely left limits and is* $P$*-almost surely right continuous.*

For a given $\left( \theta,a,o \right)\in\Theta\times E\times M$ the signal process is a Lévy process denoted $X\left( t;\theta,a,o \right)$.

The characteristic function of any such process is completely described by a triplet $\left( C,\gamma,\nu\right)$ where $\gamma$ is the drift, $C$ the Gaussian covariance matrix and $\nu$ the Lévy measure. We assume that the total mass of the measure $\nu$ is uniformly bounded:

(S-34)

${sup}_{\theta,a,o} \nu\left( R^{d};\theta,a,o \right)<\infty$

From (S-34) we know that for any state, option and control the jumping times are almost surely at most countable and in increasing order, exponentially distributed with rate $\frac{1}{\nu\left( R^{d} \right)}$. We also know that the process is the independent sum of a drift diffusion process and a Compound Poisson Process $\left( CPP \right)$. We write that $X(t)_{t\geq0}$ is a $CPP\left( \lambda,F \right)$ with rate $\lambda\in R_{+}$and probability distribution $\mu$ on $R^{d}$ if

(S-35)

$X\left( t \right)=\sum_{j=0}^{N\left( t,\lambda\right)} Y_{j}$

where $N\left( t,\lambda\right)$ is the Poisson process with rate $\lambda t$ and the random variables $Y_{j}$ are independent identically distributed as $\mu=\frac{\nu}{\nu\left( R^{d} \right)}$.

***S-3.1 Information Decay***

The leaky process defined by (7) is not a Lévy process even if $X(t)_{t\geq0}$ is: this is going to be clear after we consider its characteristic function. However we can derive important information from what we know of the underlying process. We recall that if $X(t)_{t\geq0}$ is a Lévy process, then its characteristic function has a simple form, entirely determined by the value $X\left( 1 \right)$. More precisely, there is a function $\eta_{X}:R^{d}\to C$ such that

(S-36)

$Ee^{i\left( u,X\left( t \right) \right)}=e^{t\eta_{X}\left( u \right)}$

We can then derive the characteristic function of the leaky process:

**Proposition 16** *Let* $LX$ *be define by equation (7), and* $\eta_{X}$ *be the characteristic exponent of* $\left( X(t)_{t\geq0} \right)$*. Then for every* $u\in R^{d}$ *:*

(S-37)

$Ee^{i\left( u,LX\left( t \right) \right)}=e^{\int_{0}^{t} \eta_{X}\left( ue^{-\rho\left( t-s \right)} \right)ds}$

We illustrate the evidence gathering leaky process in two simple examples, applying the formula in (S-37) for the characteristic function.

***S-3.2 Pure drift diffusion process***

Take a one dimensional process $\left( d=1 \right)$, with $C=\sigma^{2}$, constant independent of the triple $\left( \theta,a,o \right)$, and drift of the form $\gamma\left( \theta,a,o \right)=g\left( a \right)u\left( \theta,o \right)$, where $g$ is an increasing function. Thus, the drift is proportional to the utility of the option in the true state. Equation (S-37) gives in this case:

(S-38)

$\eta_{X}\left( v \right)=i\left( v,\gamma\right)\frac{1-e^{-\rho t}}{\rho}-\frac{1}{2}\sigma^{2}v^{2}\frac{1-e^{-2\rho t}}{2\rho}$

$((v,\gamma)$ is the inner product $)$. The leaky process at time $t$ for any attention path $e(s;a)_{s\geq0}$ to option $a\in M$ is a normal random variables with mean equal to:

(S-39)

$u\left( \theta,o \right)G\left( t,o;a \right)$

where:

(S-40)

$G\left( t,o;a \right)\equiv\int_{0}^{t} e^{-\rho\left( t-s \right)}g\left( a\left( s,o \right) \right)ds$

and variance:

(S-41)

$\frac{\sigma^{2}}{2\rho}\left( 1-e^{-2\rho t} \right)$

these are well known properties of the $OU$ process.

To summarize, the evidence gathering with decay in this case is very simple. The informative signal is the sum of two components:

1. A mean proportional to the utility in the true state of the option, times a factor under control of the $DM$, which is the weighted average (larger weight at rate $\rho$ to more recent observations) of the attention devoted until $t$ to option $a$.
2. A zero-mean term (that is independent of the option, the effort and the state) equal to:

(S-42)

$\int_{0}^{t} e^{-\rho\left( t-s \right)}\sigma dW\left( s \right)\sim N\left( 0,\frac{\sigma^{2}}{2\rho}\left( 1-e^{-2\rho t} \right) \right).$

***S-3.3 Compound Poisson Process***

The second component of our signal processes is a $CPP\left( \lambda,\mu\right)$ with mean:

$m\equiv E_{\mu}Y$

In this case

(S-43)

$\eta_{X}\left( v \right)=\lambda\left( E_{\mu}e^{i\left( v,Y \right)}-1 \right)$

Using equation (S-37) and (S-43) we can derive:

**Proposition 17** *For a* $CPP\left( \lambda,\mu\right)$*, the characteristic function of the leaky process* $LX(t)_{t\geq0}$*, is given by equation (S-37) with*

(S-44)

$\int_{0}^{t} \eta_{X}\left( ue^{-\rho\left( t-s \right)} \right)ds=\frac{\lambda}{\rho}\sum_{k=1}^{+\infty} \frac{i^{k}}{k!}\frac{m_{k}\left( u \right)}{k}\left( 1-e^{-\rho kt} \right)$

*where for every integer* $k$ *:*

(S-45)

$m_{k}(u)\equiv E_{\mu}(u,Y)^{k},$

*the crude moments of* $\left( u,Y \right)$*.*

To illustrate the equation (S-44) we may consider the one-dimensional case $\left( d=1 \right)$, which is the most natural for our purposes: in this case the log of the characteristic function of the leaky process in the limit as $t\to+\infty$ is

(S-46)

$\frac{\lambda}{\rho}\left( \sum_{k=0}^{+\infty} \frac{(iu)^{k}}{k!}\frac{E_{\mu}Y^{k}}{k}-1 \right).$

where the first term in parentheses is similar to the log of the characteristic function of $\mu$, but where the crude moments are divided by the factor $k$.

A second natural illustration is provided by the Poisson process, considered next.

**S-3.3.1 Leaky Poisson Process**

In this case, $\mu=\delta_{1}$. We call $L_{LX\left( t \right)}$ the distribution of $X\left( t \right)$. In this case one can prove:

**Proposition 18**

*1. The limit distribution* ${lim}_{t\to\infty} L_{LX\left( t \right)}$ *of leaky Poisson process is such that:*

1. *The* $log$ *of the characteristic function is given by* $\frac{\lambda}{\rho}f\left( iu \right)$*, with the function* $f:C\to C$ *defined by:*

(S-47)

$f\left( z \right)=\sum_{k=1}^{+\infty} \frac{z^{k}}{k!}\frac{1}{k}$

*(b) The limit distribution only depends on the parameters* $\lambda$ *and* $\rho$ *only through the ratio* $\frac{\lambda}{\rho}$*;*

*(c) All centered moments* $m_{k}\equiv{lim}_{t\to\infty} E\left( (LN(t)-E(LN(t)))^{k} \right)$ *are given by equation (S-51) below;*

*(d) In particular, the mean is equal to* $\frac{\lambda}{\rho}$*, the variance is* $\frac{\lambda}{2\rho}$*, the centered third moment is* $\frac{\lambda}{3\rho}$*. Thus, the distribution of the limit is not normal.*

*2. For every fixed* $t$ *and* $\lambda$*, the limit distribution* ${lim}_{\rho\to0} L_{LX\left( t \right)}$ *is the distribution of the Poisson process at* $t$*, and* ${lim}_{\rho\to\infty} L_{LX\left( t \right)}=0$*;*

*3.* ${lim}_{t\to0} LX\left( t \right)=0$*, the limit distribution* ${lim}_{t\to0} L_{\frac{LX\left( t \right)}{t}}$ *is the distribution of the Poisson process at t,*

Figure S-1 shows the shape of the density function for different values of $\rho$. The figure in particular illustrates that this density is, for all values of $\rho$, different from the normal. From corollary 18 we know that even the limit in $t$ is different from the normal. For example, the leaky Poisson is always skewed, and all the centered moments are positive (as one can see from equation (S-52) in the proofs section).

However, for values of $\frac{\lambda}{\rho}$ sufficiently larger than 1, a normal approximation is reasonably good. These are the ranges of $\lambda$ (large) and $\rho$ (small) that are relevant for our application to information processing of rewards. At the other extreme, as $\rho$ becomes very large, the evidence available at any time is closer to the weighted sum of the most recent Poisson spikes: the last panel of figure S-1 illustrates this point. In formulating predictions of the model for our experimental design, we will focus on the continuous paths component.

**Fig S-1.** Density function of the leaky Poisson *LN* (*t*), for *t* = 10, *λ* = 1 and various values of *ρ* as indicated. The red line indicates the density of the normal distribution with the same mean and variance. *y*-axis: number of values per million.


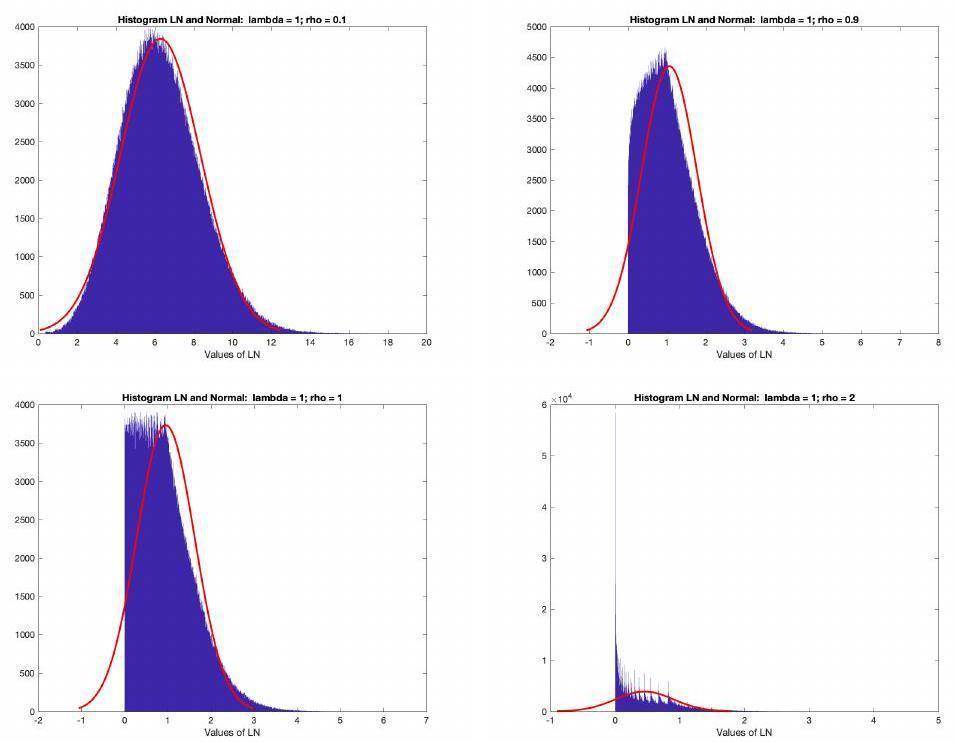


**Proof of proposition 16**

Take any partition of the interval $\left[ 0,t \right]$ into sub-intervals with extreme points $\left( s_{j} \right)_{j=0,\ldots,N-1}$, and denote $LX^{N}$ the process defined by:

(S-48)

$LX^{N}\left( t \right)\equiv\sum_{j=0}^{M} e^{-\rho\left( t-s_{j} \right)}\left( X\left( s_{j+1} \right)-X\left( s_{j} \right) \right)$

then

$Ee^{i\left( u,LX^{N}\left( t \right) \right)} =Ee^{i\left( u,\sum_{j=0}^{M} e^{-\rho\left( t-s_{j} \right)}\left( X\left( s_{j+1} \right)-X\left( s_{j} \right) \right) \right)} =E\Pi_{j=0}^{N}\left( e^{i\left( u,e^{-\rho\left( t-s_{j} \right)}\left( X\left( s_{j+1} \right)-X\left( s_{j} \right) \right) \right)} \right) =\Pi_{j=0}^{N}E\left( e^{i\left( ue^{-\rho\left( t-s_{j} \right)},\left( X\left( s_{j+1} \right)-X\left( s_{j} \right) \right) \right)} \right) =\Pi_{j=0}^{N}E\left( e^{i\left( ue^{-\rho\left( t-s_{j} \right)},\left( s_{j+1}-s_{j} \right)X\left( 1 \right) \right))} \right) =\Pi_{j=0}^{N}e^{\eta_{X}\left( ue^{\rho\left( t-s_{j} \right)} \right)\left( s_{j+1}-s_{j} \right)}$

and therefore letting the grid size $\delta_{N}\equiv{max}_{j} \left( s_{j+1}-s_{j} \right)$ of the partition tend to 0 , equation ($S-48)$ follows.

**Proof of proposition 17**

Using (S-43):

$\int_{0}^{t} \eta_{X}\left( ue^{-\rho\left( t-s \right)} \right)ds =\int_{0}^{t} \lambda\left( E_{\mu}e^{i\left( v,Y \right)}-1 \right))$

$=\lambda\int_{0}^{t} \left( \sum_{k=1}^{\infty} \int_{R^{d}} \frac{\left( i\left( u,x \right)e^{\rho\left( t-s \right)} \right)^{k}}{k!}dF\left( x \right) \right)$

$=\lambda\sum_{k=1}^{\infty} \left( \int_{R^{d}} \frac{(i(u,x))^{k}}{k!}dF\left( x \right)\int_{0}^{t} e^{-\rho k\left( t-s \right)}ds \right)$

$=\frac{\lambda}{\rho}\sum_{k=1}^{+\infty} \frac{(i)^{k}}{k!}\int_{R^{d}} (u,x)^{k}dF(x)\left( \frac{1-e^{-\rho kt}}{k} \right)$

**Proof of proposition 18**

We use equation (S-49). In this case:

(S-49)

$\int_{0}^{t} \eta_{X}\left( ue^{-\rho\left( t-s \right)} \right)ds=\frac{\lambda}{\rho}\sum_{k=1}^{+\infty} \frac{(iu)^{k}}{k!}\frac{1}{k}\left( 1-e^{-\rho kt} \right)$

Note that

(S-50)

$f\left( 0 \right)=0,f^{'}\left( z \right)=\frac{e^{z}-1}{z}$

Clearly from equation $S-49$ the limit distribution only depends on the ratio $\frac{\lambda}{\rho}$. In particular the centered moments are given by:

(S-51)

$e^{\frac{\lambda}{\rho}\left( \sum_{k=2}^{\infty} \frac{(iu)^{k}}{kk!} \right)}=\sum_{k=2}^{\infty} \frac{(iu)^{k}}{k!}m_{k}$

Note that the fourth moment is

$m_{4}=\frac{\lambda}{4\rho}\left( 1+\frac{\lambda}{\rho} \right)$

in general one can see that:

(S-52)

$\forall k:m_{k}\geq\frac{\lambda}{k\rho}$
