# Supplementary material for: Working memory and attention in choice
Source: PLoS One. 2023 Oct 11;18(10):e0284127. doi: 10.1371/journal.pone.0284127 (PMC10566694; doi:10.1371/journal.pone.0284127)
Supplement: S4 File — (DOCX) [file pone.0284127.s004.docx]

**S-4 Methods of FMRI data analysis**

***S-4.1 GLM Design***

The GLM model is described in detail in the table S-1 below. In the table, the term Dirac refers to a spike function (a probability mass of 1 at a point in time); the convolution of a Dirac with the Hemodynamic Response Function $\left( HRF \right)$ The $HRF$ is the function which is used to model the time profile of the hemodynamic response. We use, as standard, a double-gamma function. function produces an $HRF$ function beginning at that time. See section on *Whole-Brain Analysis* in the main text for more details.

The first set of predictors (*Presentation of first option*) measures the activity proportional to the event of the first presentation (intercept) and that proportional to the value of the first option. The second set (*First Inter-stimulus interval*) measures the activity occurring between the two Offers. The first predictor in the set is a sequence of Dirac measures (spike functions) at intervals of $500 ms$, beginning $500 ms$ after the first option disappears from the screen. The choice of the sequence of Dirac measures instead of a box-car is motivated by the fact that an event-related analysis may provide a more accurate model of the hemodynamic response than an epoch-based analysis.

Since we conjecture that activity declines over time, we introduce as one of the predictors *Time modulation*, linearly increasing from the beginning of the ISI to its end. The last predictor in this set is the interaction between time modulation and subjective value of the first option. The third set of predictors (*Presentation of second option*) is similar to the first set, but we add a variable measuring the value of the first option. The fourth set (*Second Inter-stimulus interval*) is simpler than the second, because we do not anticipate significant storage of memory of the value of the second option. At the moment of presentation of the two options (*Simultaneous Presentation of the two options*) we control for the event and the subjective value of the chosen option. The last set controls for head movements.

**Table S-1. GLM design** List of Predictors. *s_i_*, *i* = 1*,* 2 denotes the time of presentation of the option *i*, denoted *O_i_*. *s*_3_ denotes the time of the joint presentation. *SV* (*O_i_*) the subjective value of *O_i_*, *SV* (*Chosen*) the subjective value of the chosen option

|  | **1: Presentation of first option** |
| --- | --- |
| 1.1 | Dirac at $s_{1}$ |
| 1.2 | Dirac at $s_{1}\times$ subjective value $O_{1}$ |
|  | **2: First Inter-stimulus interval** |
| 2.1 | Train of Diracs between $s_{1}$ and $s_{1}$+4 s and $s_{1}$+4 s/6 s depending on the jitter |
| 2.2 | Train of Diracs $\times$ Time modulation |
| 2.3 | Train of Diracs $\times SV\left( O_{1} \right)$ |
| 2.4 | Train of Diracs $\times$ Time modulation $\times SV\left( O_{1} \right)$ |
|  | **3: Presentation of second option** |
| 3.1 | Dirac at $s_{2}$ |
| 3.2 | Dirac at $s_{2}\times SV\left( O_{1} \right)$ |
| 3.3 | Dirac at $s_{2}\times SV\left( O_{2} \right)$ |
|  | **4: Second Inter-stimulus interval** |
| 4.1 | Train of Diracs between $s_{2}$ and $s_{2}+4s/6s$ depending on the jitter |
| 4.2 | Train of Diracs $\times$ Time modulation |
|  | **5: Simultaneous Presentation of the two options** |
| 5.1 | Dirac at $s_{3}$ |
| 5.2 | Dirac at $s_{3}\times SV($ Chosen $)$ |
|  | **6: Choice** |
| 6 | Dirac at motor response at $s_{3}$+ response time |
|  | **7: Motion corrections** |
| 7 | Movement (6 reg, 1 value/volume) and |
|  | Movement ${}^{2}$ (6 reg. 1 value/volume) |

***S-4.2 Finite Impulse Response (FIR)***

The Finite Impulse Response *FIR* analysis gives us insight on the pattern over time of the BOLD response to a stimulus. Once a region has been identified in the whole brain analysis, the study of the time path can provide essential additional information on the time path of the brain response in that region. In the following we shorten "presentation of option" to "offer".

The BOLD signal is typically assumed to be a linear function of the explanatory variables. The effect of the explanatory variables however is not immediate, but instead occurs as a linear combination of past contributions; so the explanatory variables are introduced in the regression as a convolution of the stimulus function and an impulse response. Given that the precise form of the impulse response (in particular its dependence on brain regions and individuals) is not known, the analysis has to allow variability in the shape of the response. A convenient way to achieve this is to represent the impulse function as combination of Temporal Basis Functions $\left( TBF \right)$. A convenient choice of $TBF$s is boxcars (indicator of an interval) or Dirac delta functions; in this case the basis functions are Finite Impulse Responses $\left( FIR \right)$. This is the representation we choose here. We also allow modulation of the basis delta functions by the subjective value of the options. In summary, the BOLD response is represented as a weighted sum of finite impulses in the past, each $k^{th}$ impulse occurring with a finite lag $\tau_{k}$. The weights are the coefficients to be estimated; the weight for a lag $\tau_{k}$ represents the contribution of an impulse occurring $\tau_{k}$ units of time in the past; each weight may depend on the subjective value, and so is the sum of an intercept depending only on $k$ and of the product of another $k$ dependent weight times the $SV$ of the option being considered.

To obtain the event-related average BOLD for each stimulus of interest, we chose the $FIR$ as the basis function to model the BOLD response in a second GLM. The FIR set consists of a number of successive post-stimulus time bins, and its implementation in a $GLM$ effectively averages the BOLD response at each post-stimulus time. The BOLD path was extracted from clusters of approximately 27 voxels, obtained by creating a $3\times3\times3$ box centered on the voxel with the largest $z$ statistics in the ROI's identified in the GLM parametric analysis. We denote $t$ time variable, $n$ trial variable, $t_{0}^{n}$ starting time of trial, $sv^{n}$ subjective value in trial $n$. The variable $k=1,\ldots,K$ is the time index (time unit $600 ms$ ). The index $i$ of participant's id, and voxel index are not indicated.

The precise equation for the BOLD response is in equation (S-53) below, where $y^{n}\left( t-t_{0}^{n} \right)$ is the value of the BOLD in trial $n$ at time $t-t_{0}^{n}$ after the onset of the relevant event (for example, after the presentation of the first offer, or the second), and $\delta_{k}\left( t-t_{0}^{n} \right)=1$ if $k=t-t_{0},=0$ otherwise, so the $k^{th}$ term $\beta_{k}+\gamma_{k}sv^{n}$ is non zero if and only if $t-t_{0}^{n}=k$ :

(S-53)

$y^{n}\left( t-t_{0}^{n} \right)=\alpha+\sum_{k=0}^{K} \delta_{k}\left( t-t_{0}^{n} \right)\left( \beta_{k}+\gamma_{k}sv^{n} \right)+\epsilon$

(see also section $S-4.2$ below for details). If the $\gamma_{k}$ weights are set to 0 then the $\beta_{k}$ coefficient is approximately the average of the BOLD signal $k$ time units ago. In the complete model in equation (S-53) above, the component represented by the term $\gamma_{k}sv^{n}$ gives an estimate of the additional contribution to the $BOLD$ from the subjective value of the trial $sv^{n},k$ time units ago. In the complete model the weight $\beta_{k}$ is approximately the average of the BOLD at the $k^{th}$ time unit, minus the $\gamma_{k}$ coefficient times the average over the entire session of the subjective value. At the moment of presentation of Offer 2 we estimate the modulation of BOLD signal by both offer 1 and offer 2 , so we estimate the model S-54.

(S-54)

$y^{n}\left( t-t_{0}^{n} \right)=\alpha+\sum_{k=0}^{K} \delta_{k}\left( t-t_{0}^{n} \right)\left( \beta_{k}+\gamma_{k}^{1}sv_{n}^{1}+\gamma_{k}^{2}sv_{2}^{n} \right)+\epsilon$

The standard method of course is obtained setting $\gamma_{k}=0$ for every $k$.

***S-4.3 Functional Connectivity***

In an experiment with $n=1,\ldots,N$ trials, a set of stages $s=1,\ldots,S$ occurs at time $t_{s}^{n}$. In our experiment, two stages, presentation of first and second offer, occur in each trial. The beta-series method estimates, for each trial $n$ and each stage $s$ within the trial, a beta coefficient for the variable $X_{s}^{n}$ equal to the convolution of the Dirac $\delta_{t_{s}^{n}}$, where $t_{s}^{n}$ is the time at which stage $s$ occurs in trail $t^{n}$ with a standard $HRF$.

To estimate the beta-series, a new model was run in $AFNI$ in a whole-brain analysis. We had 54 trials, and two stages in each trial (onset of first and second offer) so we had overall 108 separate independent variables that were introduced as separate regressors in an initial GLM. This GLM included also a regressor to model the average response to the choice screen, and six regressors of no intrinsic interest to model head motion. Subjective value was not used in this analysis. The resulting 108 parameter estimates were collected for each participant as a series of beta-values $\left( \beta_{s,t}^{v} \right)_{s=1,2,t=1,\ldots,54}$ for each voxel $v$. The beta series then provided the basis for a functional connectivity analysis, by computing for each voxel $v$ the correlation $Corr\left( \beta_{1}^{v},\beta_{2}^{v} \right)$. The assumption of the method is that the regions whose beta-series are more correlated during a certain event of interest (for example, the second offer) are considered to be more functionally interacting for that event. The anatomically defined bilateral $vmPFC$ was used as the seed region for our functional connectivity analysis. This area was chosen because the whole-brain analysis revealed its significant activation at the display of the second offer, confirming its well-known role in representing decision-values.
