# Supplementary material for: Working memory and attention in choice
Source: PLoS One. 2023 Oct 11;18(10):e0284127. doi: 10.1371/journal.pone.0284127 (PMC10566694; doi:10.1371/journal.pone.0284127)

**S-5 Additional Behavioral Analysis Results**

**Fig S-2. Distribution of log RT.**


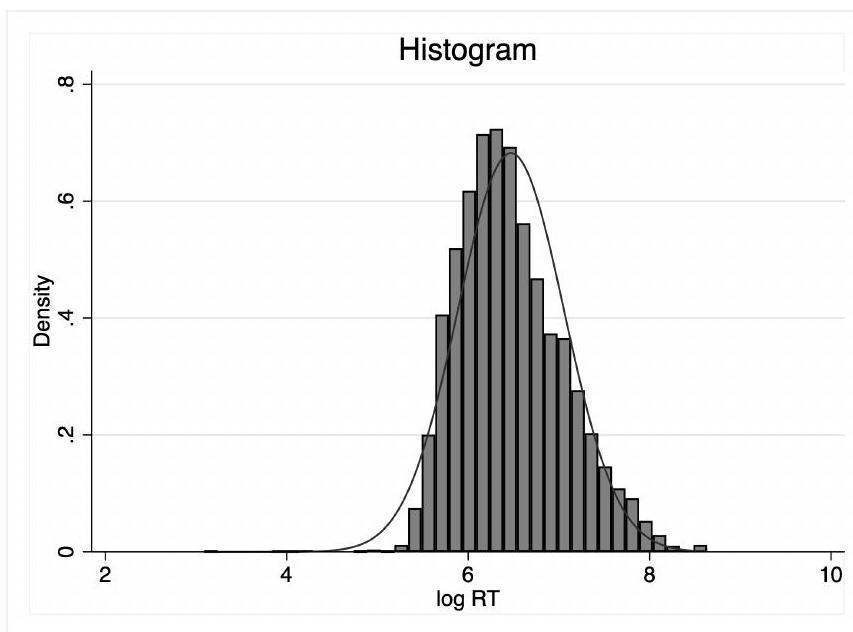


**Fig S-3. Test of normality of log RT.**


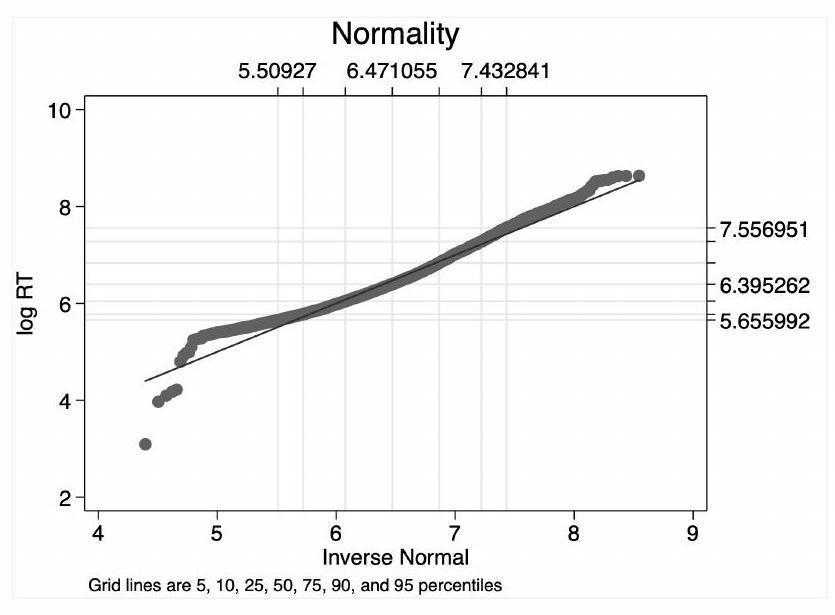

Supplement: S5 File — (DOCX) [file pone.0284127.s005.docx]
