# Supplementary material for: Working memory and attention in choice
Source: PLoS One. 2023 Oct 11;18(10):e0284127. doi: 10.1371/journal.pone.0284127 (PMC10566694; doi:10.1371/journal.pone.0284127)
Supplement: S6 File — (DOCX) [file pone.0284127.s006.docx]

**S-6 Additional FMRI Results**

In this section we report the details of the results of our analysis of $fMRI$ data.

***S-6.1 Activation at Second Offer***

**Table S-2. Region with activation proportional to the subjective value of the second offer** (variable 2.2 in Table S.4.1). Images thresholded using clusters determined by *Z >* 3*.*2, (*p <* 0*.*001) and a (corrected) cluster significance threshold of *p* = 0*.*05.

| Voxels | P | Z-max | x | y | z | *Location of peak voxel* |
| --- | --- | --- | --- | --- | --- | --- |
| 2288 | 4.17 e-07 | 4.44 | -48 | 14 | 40 | Middle Frontal Gyrus |
| 1243 | 8.12 e-05 | 5.11 | -38 | -76 | 36 | Left Lateral Occipital Cortex |
| 699 | 0.0024 | 4.67 | -22 | -4 | 16 | Left Putamen |
| 625 | 0.0041 | 4.84 | 44 | -68 | 42 | Right Lateral Occipital |
| 410 | 0.0207 | 4.17 | 16 | 10 | 16 | Right Caudate |
| 389 | 0.0246 | 3.95 | -28 | 54 | 0 | Left Frontal Polar |

**Fig S-4. Decaying BOLD activity during delay in *SPL*.** (Top panel) BOLD activity decreases linearly with time between the onset of offer 1 and offer 2 (variable 2.2 in Table [S-1).](#_heading=h.gjdgxs) (Bottom Panel) Correlation between BOLD activity and subjective value of offer 1 decrease with time in the delay period. (variable 2.4 in Table [S-1.)](#_heading=h.gjdgxs)


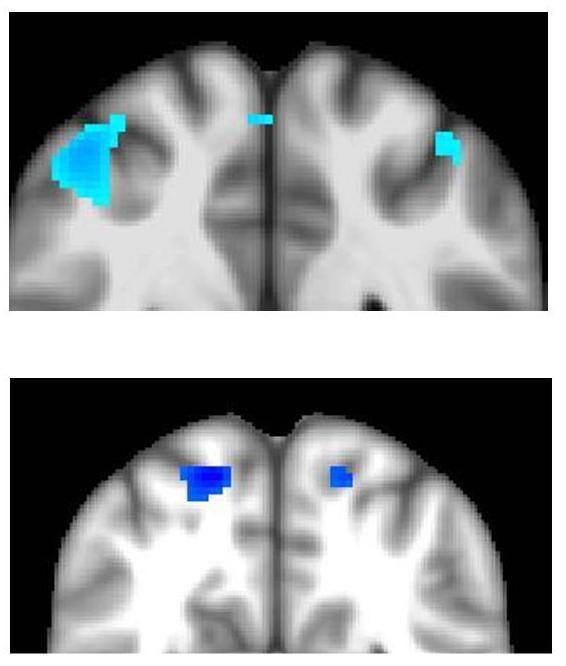


**Fig S-5. Option 2 at** *vmPFC/OMPFC***.** Parametric effect of the subjective value of offer 2 at onset of offer 2 (variable 3.3 in Table [S-1.)](#_heading=h.gjdgxs) Cross point at (*x, y, z*) = (28*,* 54*,* 0). Cluster displayed with estimated significance at *p*-value = 5 % *FWE* corrected clusterwise.


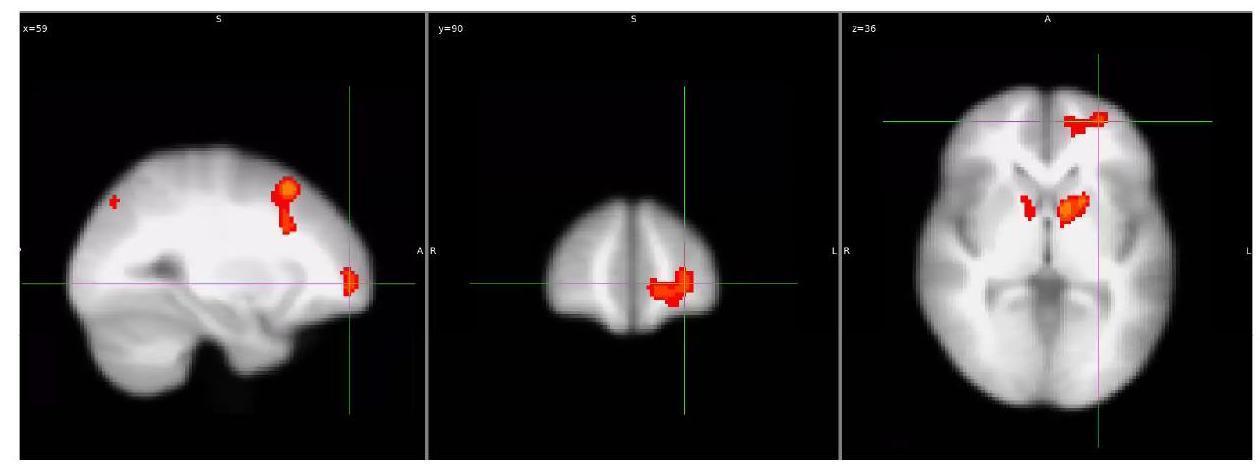


***S-6.2 FIR analysis***

Figure S-6 reports the results of the $FIR$ analysis of activation in reward areas, after presentation of the first and second offer.

**Fig S-6. Parametric modulation of BOLD signal in** *SPL* **by the SVO1 and SVO2 at presentation of Offer 1 and Offer 2.** Left panel: effect of SVO1 at Offer 1. Middle panel: effect of SVO1 at Offer 2. Right panel: effect of SVO2 at Offer 2. Bars are 95 % confidence intervals.



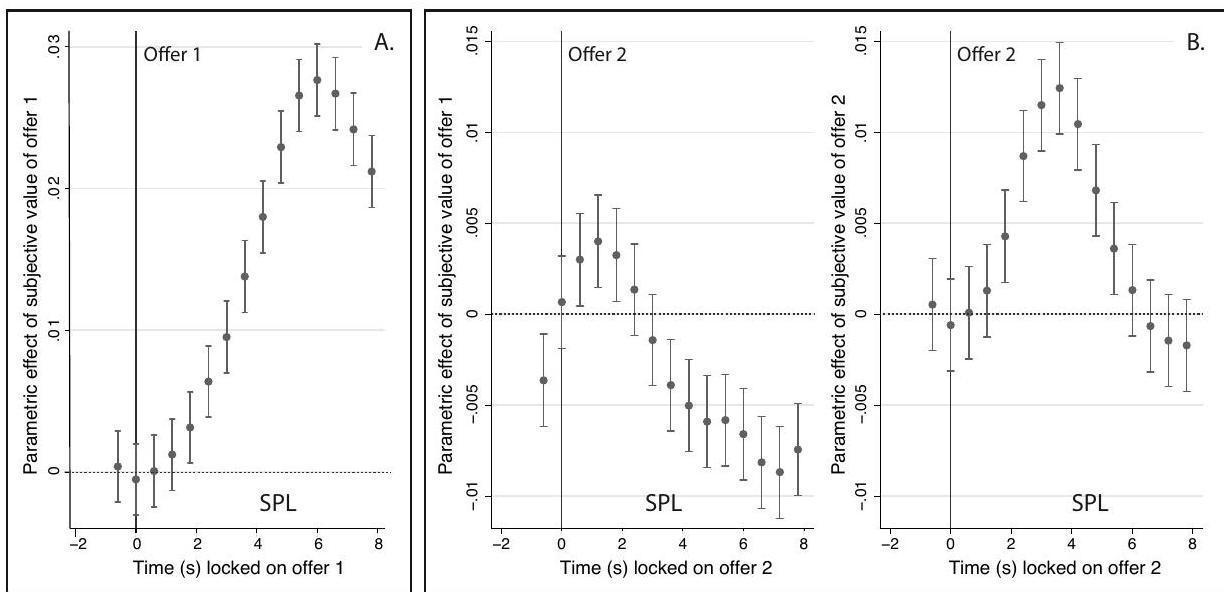


***S-6.3 Reverse Inference***

Figure S-7 reports the comparison between two network of regions: one recording the decline in activity after the presentation of the first option (corresponding to the reduction in the value stored for that option), and the other describing areas associated to the term working memory in reverse inference analysis in Neurosynth.

**Fig S-7. Comparison of reverse inference meta analysis and activation** Left panel: *Neurosynth* regions associated with working memory. Reverse inference based on 1091 studies. Right panel: regions where BOLD activity decreases linearly with time between the onset of offer 1 and offer 2 (variable 2.2 in Table [S-1).](#_heading=h.gjdgxs) Both cross-hairs are at (44*, −*46*,* 54).


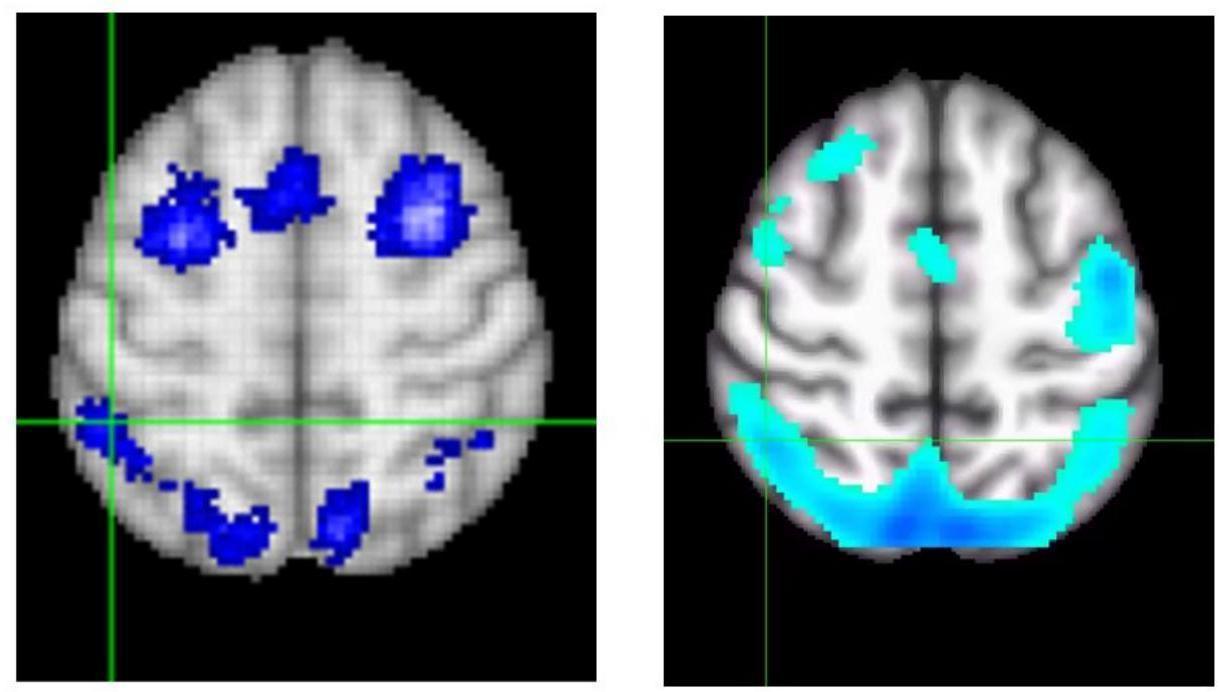


***S-6.4 Functional Connectivity***

Contrasts of correlations obtained with the Rissman method (see Methods section S-4.3) of functional connectivity with $vmPFC$ as a seed showed significantly greater functional connectivity at the moment of presentation of the second option than at the moment of presentation of the first option for multiple connected brain regions. After correction for spatial correlation using a Monte Carlo simulation-based estimator ( $AFNI$ 3 dClustSim), clusters showing stronger functional connectivity to $vmPFC$ during the presentation of the second offer contrasted with the first corresponded anatomically to superior temporal gyrus extending to left insula (BA 22), right inferior frontal gyrus (BA 45), right parietal lobe (BA 40), Left middle frontal gyrus (BA 10). No region was identified as more functionally connected to $vmPFC$ during presentation of the first offer with respect to the second.

**Table S.6.2: Functional connectivity analysis, *vmPFC* taken as seed region.** Contrast reporting correlation at the moment of presentation of second offer larger than at first offer. *p* = 0*.*0005*, q*(*FDR*) = 0*.*05. Coordinates of voxels of peak significance, *MNI*. A * under *CS* (Cluster Size) denotes significance at *α* = 0*.*05 after correction for spatial correlation (3*DClustSim* in *AFNI*).

| #Voxels | Peak x | Peak y | Peak z | CS | Anatomical region |
| --- | --- | --- | --- | --- | --- |
| 140 | -57.8 | -8.2 | 2.2 | * | Superior Temporal Gyrus |
| 139 | 54.2 | -15.2 | 2.2 | * | Right Inferior Frontal Gyrus |
| 85 | 64.8 | 37.2 | 40.8 | * | Right Inferior Parietal Lobule |
| 70 | -36.8 | -43.2 | 23.2 | * | Left Middle Frontal Gyrus |
| 65 | -1.8 | -8.2 | 51.2 |  | Left Medial Frontal Gyrus |
| 34 | 68.2 | 19.8 | 16.2 |  | Right Post-Central Gyrus |
| 31 | 33.2 | -8.2 | 61.8 |  | Right Middle Frontal Gyrus |
| 30 | -19.2 | 61.8 | -4.8 |  | Lingual Gyrus |
| 29 | 50.8 | 5.8 | 58.2 |  | Right Precentral Gyrus |
| 27 | 36.8 | -22.2 | 2.2 |  | Right Insula |
